# Genome-wide mutational spectra analysis reveals significant cancer-specific heterogeneity

**DOI:** 10.1038/srep12566

**Published:** 2015-07-27

**Authors:** Hua Tan, Jiguang Bao, Xiaobo Zhou

**Affiliations:** 1 Center for Bioinformatics & Systems Biology, Department of Radiology, Wake Forest School of Medicine, Winston-Salem 27157, USA; 2 School of Mathematical Sciences, Beijing Normal University, Beijing 100875, China; 3 College of Global Change and Earth System Science, Beijing Normal University, Beijing 100875, China

## Abstract

Cancer is widely recognized as a genetic disease in which somatic mutations are sequentially accumulated to drive tumor progression. Although genomic landscape studies are informative for individual cancer types, a comprehensive comparative study of tumorigenic mutations across cancer types based on integrative data sources is still a pressing need. We systematically analyzed ~10^6^ non-synonymous mutations extracted from COSMIC, involving ~8000 genome-wide screened samples across 23 major human cancers at both the amino acid and gene levels. Our analysis identified cancer-specific heterogeneity that traditional nucleotide variation analysis alone usually overlooked. Particularly, the amino acid arginine (*R*) turns out to be the most favorable target of amino acid alteration in most cancer types studied (P < 10^−9^, binomial test), reflecting its important role in cellular physiology. The tumor suppressor gene TP53 is mutated exclusively with the HYDIN, KRAS, and PTEN genes in large intestine, lung, and endometrial cancers respectively, indicating that TP53 takes part in different signaling pathways in different cancers. While some of our analyses corroborated previous observations, others indicated relevant candidates with high priority for further experimental validation. Our findings have many ramifications in understanding the etiology of cancer and the underlying molecular mechanisms in particular cancers.

Cancer is recognized as a disease resulting from the gradual accumulation of somatic mutations in the genome^1^,^2^,^3^,^4^. The mutations sequentially endow the selected clones with advantageous self-sufficiency in growth signals, capability of evading apoptosis and metastasizing to remote sites^5^,^6^. Continuing efforts of comprehensive sequencing over the past decade, especially genome-wide screening, have detailed the genomic landscape of cancers such as brain^7^, pancreatic^8^, breast and colorectal^9^, and bladder^10^; pediatric cancer^11^; and rare types such as gingival buccal oral carcinoma^12^. These efforts have focused on specific cancer types and identified up to hundreds of somatic mutations in a given tumor. Some genes are altered in many cancer types, while others exemplify strong cancer specificity. Despite much work, the underlying mutational spectra of a particular cancer, as well as its difference from other cancers, remains to be clarified.

A comprehensive investigation of genetic mutations of various cancers based on integrative data sources would help to identify mutational spectra in a cancer-specific manner. Some previous studies did address patterns of somatic mutations in human cancer genomes^13^,^14^. These researches focused on patterns of DNA base pair changes and were restricted to very few cancer types (e.g., lung, breast and colorectal). Other work emphasized a general census of human cancer genes and potentially related signaling pathways, but did not provide detailed mutational profiles of these cancer genes^15^,^16^, or their cancer specificity. Very recently, integrative analyses have identified recurrent genetic aberrations of particular cancers, such as glioblastoma^17^ and oral squamous cell carcinoma^18^. These studies represented a valuable attempt towards integrating existing resources for new discoveries of mutational spectra, but were not comprehensive comparative studies across cancer types.

The most integrative analyses on mutational heterogeneity so far were done by Lawrence and colleagues^19^, and Alexandrov and colleagues^20^,^21^. These important studies explored the heterogeneous mutational signatures in the cancer genome across different cancer types, which is significant for understanding the correlation between genomic evolution and environmental exposures for different cancers. But these efforts were limited to nucleotide base pair changes, without further investigating the associated amino acid substitutions and the genes carrying those somatic mutations. This issue is essential since an amino acid is encoded by a nucleotide triplet, and hence one base pair change may lead to several possible amino acid substitutions, which eventually results in distinct biochemical/biophysical properties of related proteins^22^ that vary with cancer types. On the other hand, the 12 base pair changes (A, T, G and C may change to any of the remaining three nucleotides) generally maintain distinct profiles in different cancer genes. Therefore, counting the total numbers of each base pair change in a tumor sample is uninformative for inferring the significant cancer genes. Further analyzing amino acid residue substitutions and mutation frequency of related cancer genes would provide additional insights into the heterogeneity of mutational spectra.

In the present study, we extracted and systematically analyzed more than one million non-synonymous mutations from the latest Catalogue of Somatic Mutations in Cancer^23^ (COSMIC v68). Our investigation involved ~8,000 genome-wide screened samples across 23 major human cancers and about 20,000 genes. We conducted analyses using the genome-wide association study (GWAS) approach, a powerful tool to study associations between molecular traits and particular phenotypes^24^,^25^,^26^,^27^. Specifically, we explored the general mutational signatures of various cancer types, compared the most frequently mutated genes in different cancers, and investigated the mutational landscape at the amino acid level. Since the current COSMIC database has now incorporated information of patient age, we analyzed potential correlations between mutation occurrences and patient age at diagnosis.

We also tested the hypothesis about combinatorial mutational patterns of gene pairs, one mutually exclusive and one co-mutational^28^. These two patterns indicate whether (exclusive pattern) or not (co-mutational pattern) the associated genes are likely to function in the same signaling pathway^1^,^29^,^30^. Therefore, identifying gene pairs of particular combinatorial mutational patterns with high statistical significance has considerable biological meaning, particularly for inferring oncogenic network modules for a specific cancer^30^. The current COSMIC database contains a number of mutations from genome-wide screened clinical samples, which provides a unique opportunity to systematically test the combinatorial pattern hypothesis with an enhanced statistical approach. Our results recapitulated many previous observations and also detected novel candidates of gene pairs with high statistical significance.

## Results

### General mutational landscape of various cancer types

**Figure 1:**
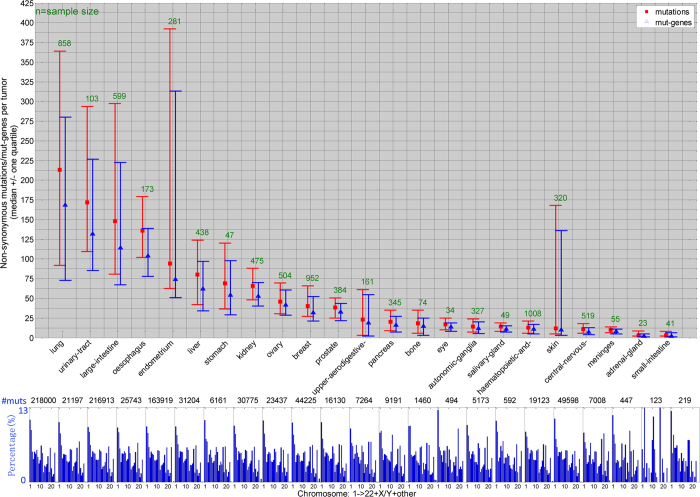
Number of non-synonymous somatic mutations and mutated genes per tumor in major human cancers. Mutations were detected by genome-wide sequencing studies curated from the COSMIC database (v68). Squares and triangles indicate median of the number of mutations and mutated genes, respectively; horizontal bars stand for the 25 and 75% quartiles. The positive integer above each bar represents number of genome-wide screened samples of that cancer. The lower panel subgraphs illustrate distribution of mutations along the chromosomes for individual cancer types, with the order identical to the *x*-labels of the upper panel, including 22 autosomes and two sex chromosomes denoted as *X* and *Y*. Undentifiable chromosomes are denoted as *other*. The number of missense mutations for each cancer is presented above each subgraph.

In the current COSMIC database, the average number of missense mutations and mutated genes per tumor sample varied dramatically with cancer tissues (Fig. 1). Lung, urinary tract, and large intestine cancers displayed more than 150 missense mutations involving up to 100 protein-coding genes per tumor sample. Other types, such as central nervous system and meningeal cancers, typically contained fewer than ten somatic mutations. Sample variations within a particular cancer type also existed (deviation bars in Fig. 1, upper panel). In general, tissues that divide rapidly and self-renew frequently, such as endometrium, ovary, and liver, tended to bear more somatic mutations than those that do not. In addition, tissues frequently exposed to external carcinogens from food, air, or ultraviolet light (e.g. esophagus, lung, and skin cancers), possessed significantly more mutations than others, consistent well with the previous molecular epidemiology studies of human cancers^31^ and genome-wide statistical analysis studies^15^,^19^.

**Figure 2:**
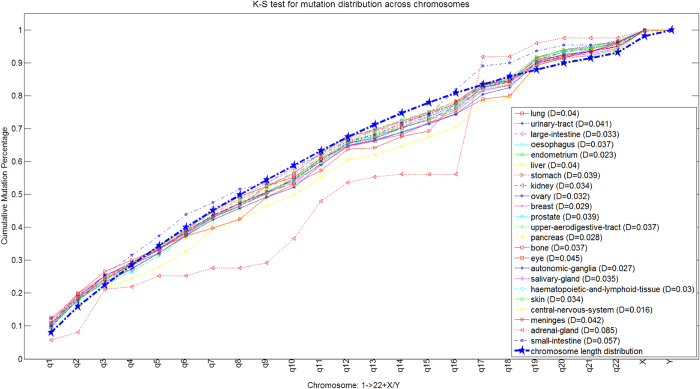
Kolmogorov-Smirnov test results for distribution of mutations across chromosomes for 23 major human cancers. Somatic mutations of all cancers have a similar frequency of distribution to chromosome lengths (D < 0.05), except for adrenal gland and small intestine cancers.

We are interested in whether these mutations occurred preferentially in particular chromosomes of the whole genome. Thus, we explored distributions of somatic mutations across the 23 chromosomes for each cancer type. Distribution of mutations across chromosomes for 23 human major cancers are illustrated by ‘rainfall’ plots (supplementary Figures S1-S23). In general, the longer the chromosome, the more mutations could be detected. To test this correlation quantitatively, we applied the Kolmogorov-Smirnov test to determine differences between mutation distribution and chromosomal length (Methods). All cancers except adrenal gland and small intestine showed no clear chromosomal preference for the mutations (Fig. 2). For example, for lung cancer, there were more mutations than expected at q1-q3 and q19-q22, and fewer at q9-q10 and q13-q18; but overall, the difference was not statistically significant (the K-S statistic D < 0.05), implying negligible chromosomal preference for lung cancer mutations based on the data in the current COSMIC.

### Top frequently mutated genes in a cancer-specific sense

**Figure 3:**
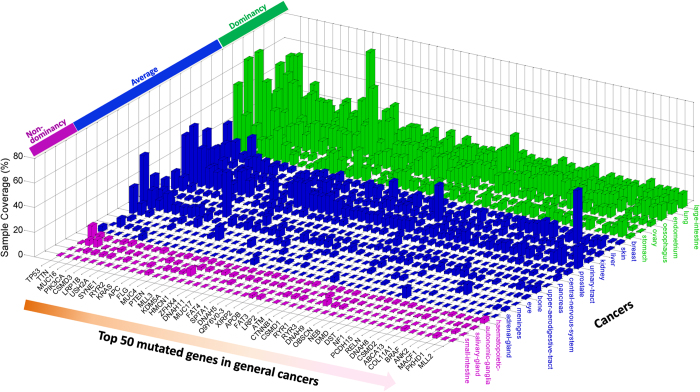
Most frequently mutated genes in general cancers. Shown are top 50 mutated genes for all cancer types detected in COSMIC v68. Cancers with at least one gene that muated in no less than 50% of the screened samples were termed ‘dominancy’ (right part); cancers with no gene mutating in more than 10% of the screened samples were termed as ‘non-dominancy’ (left part); the remainder were termed ‘average’ (middle part).

We then sorted mutated genes according to their total missense-mutation occurrences and statistical significance in human cancers (Table S2). Figure 3 shows the mutational landscape of the top 50 frequently reported genes in general 23 cancers. A list of the top 1000 genes is given in Table S2A. Most of the top-ranked genes are well-known tumor suppressor genes (TSG) or oncogenes, such as TP53, phosphoinositide 3-kinase (PIK3CA), adenomatous polyposis coli (APC), and GTPase KRas (KRAS) genes. The titin (TTN) gene was rarely recognized as a tumor-associated gene in the existing literature, but it ranked in the top 2 in the list. TTN encodes a giant protein (>30000 amino acids), which poses a high risk of residue alterations because of random DNA repair error. Another giant protein is the membrane-associated mucin (MUC16), which contains ~22000 amino acids, also ranked high in our list. From a perspective of functional classification, most missense mutations on these proteins are likely to be ‘passenger’ mutations, which would not directly confer a selective growth advantage^14^. To differentiate passengers from driver mutations is another essential task in molecular cancer research, as we have previously addressed^22^. Recently, researchers provided an insightful explanation about the frequent (but probably just passenger) mutation of these two genes^19^. Our current analysis also identified interesting patterns different from those of recognized cancer-associated genes (described below).

Mutation frequencies of some genes varied extensively between cancer types. For example, mutations in the top-ranked gene, TP53, were not reported in thyroid, soft-tissue, cervix, or parathyroid tumors in the current COSMIC database. By contrast, a relatively less frequently mutated gene, BRAF (46^th^ in the list, Table S2A), was altered in about half of skin cancers (sample coverage 43.1%), corroborating the initial screening conducted a decade ago^32^. These tumors may progress through very different mechanisms, or be activated by particular exogenous mutagens. Indeed, previous work has identified several mutagens for different cancers, e.g., sunlight-associated skin cancer, tobacco-associated lung cancer, and dietary-associated colon cancer^13^.

The top 10 frequently mutated genes and their mutation frequency for individual cancer types are listed in supplementary Table S2B. Some genes do not appear in the top 50 mutated genes for general cancers as shown in Fig. 3, since they tend to mutate predominantly in particular cancer types. Besides the most important gatekeeper gene (TP53), from the current COSMIC we detected cancer-specific frequently mutated genes that have been widely known to play critical role in tumor progression, e.g., the APC and PI3KCA genes in large intestine cancer^33^, the BRAF gene in skin cancer^32^, the KRAS gene in pancreatic cancer^34^, the VHL and PBRM1 genes in kidney cancer, and the CTNNB1 and KDM5A genes in liver cancer^35^,^36^. Interestingly, the PBRM1 and BAP1 genes were recently reported as novel targets for renal cell carcinoma^37^. Mutations in the BAP1 gene occurred in 34 out of 475 kidney samples (Table S2A), placing that gene among the top eight in the list. These studies partially verified the reliability of our comprehensive analyses.

We calculated the statistical significance of each gene based on its sample coverage and protein sequence length by binomial test (supplemental materials and Table S2C), and sorted the genes for each cancer by the *p*-values. Genes with equal *p*-values are secondarily sorted according to their mutation frequency as shown in Table S2A. The top 10 genes with smallest *p*-values are listed in Table S2D. Some cancers (6 out of 23) had a large overlap (9–10 genes overlap) with the original list determined by sample coverage alone, while others differed from the original to various extents (2–8 genes overlap). Briefly, after correcting for sequence length, the TTN and MUC16 genes did not rank the top 10 for some cancers (e.g., breast, liver, kidney, etc.) any more, implying that their high mutation frequency in these cancers was largely due to their long sequence without a statistical significance. On the other hand, TTN and/or MUC16 were still retained in the top 10 for some cancers such as large intestine and lung cancers, suggesting their tumorigenic relevance to these cancers.

Based on the mutation frequency of each mutated gene detected in the current COSMIC, mutational patterns can be roughly categorized into three types, which we termed as dominancy, non-dominancy, and the average status of the two (Fig. 3). The first class (‘dominancy’) has one or a few ‘fingerprint’ mutant genes, which mutate in over 50% of tested samples (here the percentage thresholds are not essential and the categorical terms are loose, the main purpose of this classification is to demonstrate differences in gene mutational pattern between cancer types). Representative cancers of this class include large intestine, lung, endometrium, esophagus, ovary, and stomach. The fingerprint genes generally differ between cancer types. On the other hand, most non-solid tumors, such as hematopoietic-lymphoid and autonomic ganglia cancers, have mutant genes with maximum sample coverage lower than 10% (‘non-dominancy’). For example, the most frequently mutated gene of autonomic ganglia cancer, ALK, was found to mutate in only 30 out of 327 autonomic ganglia tumor samples (sample coverage 9.2%). The remaining cancers (‘average’) locate between these two extremes, with mutant genes of maximum sample coverage ranging from 14.3% (TP53 in prostate cancers) to 47.6% (TTN in urinary tract cancers). For those with fingerprint mutants, targeting the involved genes and the point mutations might be the next step; for others, it would be more promising to consider different mechanisms such as oncogenic gene fusion^38^, copy number variation^25^ or epigenetic changes^39^.

### Mutational landscape at the amino acid resolution

**Figure 4:**
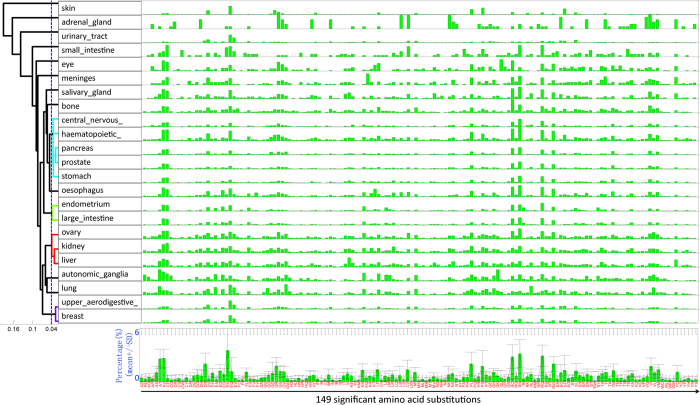
Distribution of mutation frequency along 149 significant amino acid substitutions. Each row of the upper panel corresponds to one cancer as denoted on the left, and each bar stands for the occurrence frequency of a residue substitution as represented in the bottom subfigure. All 149 frequencies of each cancer constitute its substitution spectrum; then cancers are clustered according to their similarity in substitution spectra, as shown by the left dendrogram. The lower panel shows the average substitution spectrum for all the cancers with standard deviations denoted. A higher resolution of the average substitution spectrum and the clustering dendrogram is included in supplementary Figure S26.

We analyzed amino acid substitutions from genome-wide screened samples for each cancer type and analyzed the frequency of mutations among the 380 possible amino acid changes (Methods). Some substitutions never occurred in any one cancer type, e.g., A > Q, Y > W. Others occurred in less than 1% of the mutation records of all cancer types in total, e.g., A > H, Y > V (Figure S25). Excluding these rare substitutions, we obtained 149 significantly occurring amino acid alterations. Figure 4 illustrates the distribution of mutation frequency of the 149 substitutions for 23 human cancers. The frequency distribution formed a unique mutational spectrum at the amino acid level for each cancer. We then clustered the cancers according to their spectrum (Methods). Several groups with a high degree of similarity in frequency distribution were clearly discerned (Fig. 4, dendrogram on the left upper panel). Most notably, the breast and upper aerodigestive tract cancers had nearly identical amino acid substitution spectra, dominated by the E > K mutation. This point mutation resulted from the G > A base pair change at the DNA level. Likewise, shared significantly analogous mutational patterns were found among lung and automatic ganglia cancers; ovary, kidney, and liver cancers; and endometrial and large intestine cancers.

The average frequency of mutations across all cancer types for each amino acid substitution is also illustrated in Fig. 4 (lower panel). A higher resolution of the 149 amino acid changes and the clustering dendrogram is included in Figure S26. Considering the 23 cancers simultaneously, the top 10 dominant amino acid substitutions were the E > K, R > H, R > Q, R > C, A > V, A > T, D > N, P > L, R > W, and G > R substitutions. Remarkably, all 10 amino acid substitutions can invariably be attributed to the G > A or C > T nucleotide alterations from the DNA codon table, indicating that our results are consistent with previous nucleotide variation studies^13^,^19^,^21^.

We examined the top 3 prevalent amino acid substitutions and their associated nucleotide changes for each cancer type. The most prevalent substitutions varied widely with cancer types, but most of them consisted of the aforementioned 10 dominant ones, implying that an overwhelming part of these prevalent amino acid substitutions are determined by the G > A and its dual nucleotide change C > T (i.e. on the other strand) (Table 1). This observation was further confirmed by our direct nucleotide change analysis (Figure S27), and the nucleotide mutational signature study by Alexandrov *et al.*^21^ (Table 1). Note that in Alexandrov *et al.*, the dual nucleotide changes were calculated only once, e.g., for G > A and C > T mutations, only C > T was considered. In the present study, the COSMIC database does not discriminate between DNA strands; hence, what we obtained was from both strands. We found discernable differences in dominant amino acid substitutions between cancer types, although they may exemplify identical patterns of nucleotide base pair changes. These different amino acid alterations may lead to distinct biophysical/biochemical properties in terms of hydrophobicity, polarity, charge and acidity^22^, which could be overlooked by analyzing nucleotide base pair changes alone. Most strikingly, arginine (*R*) turned out to be the most favorable target of amino acid alteration – 17 out of the 23 major cancers carries at least one arginine substitution in their top 3 amino acid substitutions (Table 1, *P* < 10^−9^, binomial test, see supplementary materials). A previous study revealed that arginine plays a pivotal role in cellular physiology, and is intimately involved with cell signaling related to tissue repair processes^40^. Our finding implies that the role of arginine in carcinogenesis also deserves investigation.

**Figure 5:**
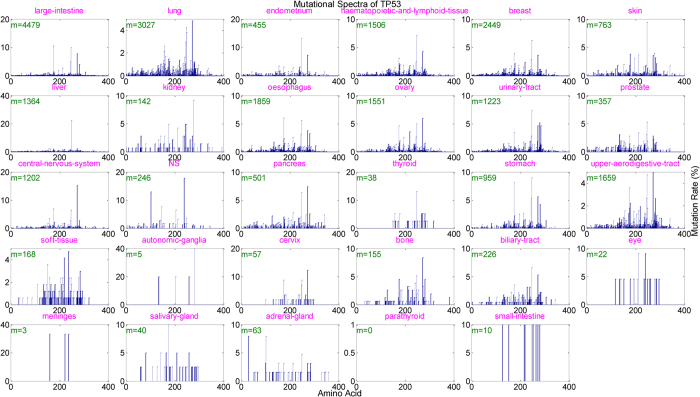
Mutational spectrum of the TP53 gene at the amino acid residue resolution. Horizontal axis represents the amino acid position along the protein sequence; vertical axis indicates the proportion of mutations in that position among all TP53 gene mutations (top left *m*’s) dected in each cancer. The mutational spectra for other top-ranked genes (KRAS, PIK3CA, PTEN, APC, TTN, and MUC16) are provided in supplementary Figures S28-S33, respectively.

To explore mutational heterogeneity along the protein sequence, we analyzed mutation site distribution across a given protein for top-ranked genes. For TP53 (Fig. 5), many positions across the whole sequence could serve as the target of residue substitution with high probability. The mutation rate varied between positions, but demonstrated some clustering properties. For instance, the region between residues 200 and 300 is the most highly mutated in various cancer types. Other highly mutated genes manifested distinct patterns. For example, in most cancer types, up to 97% of the KRAS point mutations occurred at amino acid 12 or 13, while a few mutations occurred at amino acid 61 in some cancers (Figure S28), which has been confirmed in pancreatic carcinomas^34^. The PIK3CA was frequently mutated at residue 542/545 and 1047, whereas mutations of PTEN and APC were distributed evenly at multiple sites (Figure S29-S31 respectively). Although TTN and MUC16 carried a large number of missense mutations in general, they exemplified little, if any, preference for any region of the sequences. Surprisingly, for most cancers bearing multiple TTN/MUC16 mutations (e.g., large intestine, lung, and endometrial cancers), mutation rates at all sites were invariably low (bounded by 1%; Figure S32 for TTN, Figure S33 for MUC16), very different from the well-known cancer genes discussed above.

### Correlations between occurrence of mutations and patient age

**Figure 6:**
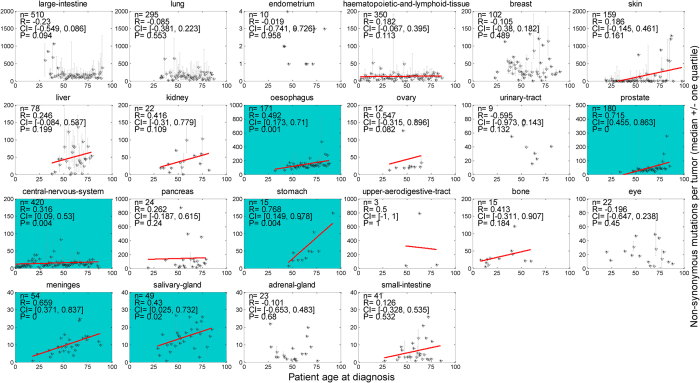
Spearman rank correlation between mutations per tumor sample and patient age at diagnosis for individual cancer types. Stars and bars stand for median and quartiles, respectively. Cancers with positive correlation coefficients are illustrated by solid fitting line, and cancers with *P* < 0.05 are encoded by dark background. *n* = number of samples; *R* = correlation coefficient; *CI* = 95% confidence interval; *P* = *p*-value.

Recent versions of COSMIC (e.g. v68) have collected patient age information for some samples, facilitating analysis of potential correlations between patient age at diagnosis and total missense mutations. We calculated the Spearman rank correlation coefficients between number of mutations and patient age, and derived the related 95% bootstrap confidence intervals (with 1000 bootstrap data samples). The correlation with *P* < 0.05 was considered significant. As shown in Fig. 6, six cancers including oesophagus, prostate, central-nervous-system, stomach, meninges and salivary-gland, displayed strong mutation-age correlation – they maintain stably increasing mutations with increasing patient age. Among these six cancers, oesophagus and stomach are typical self-renewing tissues and are susceptible to environmental mutagens before tumor initiation and during tumor progression, which results in continuing accumulation of somatic mutations in the genome^41^; while the prostate, central-nervous-system, meninges and salivary-gland cancers generally bear fewer mutations than the mutagen-exposed ones. A few cancers, such as skin, liver, kidney, ovary, bone and small-intestine, showed positive correlation between mutations and age, but not statistically significant. Most of the remained cancers demonstrated little correlation, with either small absolute correlation coefficients or too large *p*-values to be claimed as significant. Interestingly, the large-intestine cancer showed negative correlation (marginally significant, *P* = 0.094) between mutations and age, which seems counterintuitive; but patients older than 50 presented non-decreasing mutations with increasing age.

### Combinatorial mutational patterns

**Figure 7:**
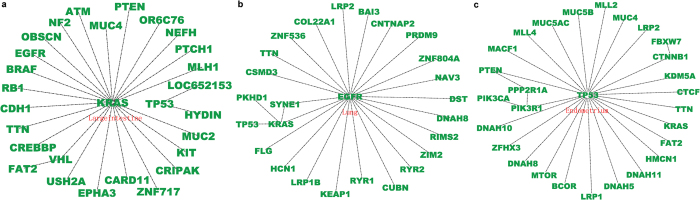
Representative gene pairs with significant exclusive pattern for three typical cancers. (**a**) large intestine cancer; (**b**) lung cancer; (**c**) endometrial cancer. Each gene pair with exclusive pattern implies that related genes tend to participate in the same cell signaling pathway (see text). The statistical analysis recapitulated the well-known gene network modules, such as the KRAS/BRAF in colon cancer, EGFR/KRAS/TP53 in lung cancer, and TP53/PTEN in endometrium. Refer to supplementary Figure S34 for the exclusive gene pairs detected in other cancers.

Two genes may tend to mutate simultaneously or in a mutually exclusive manner in a tumor sample. These combinatorial patterns have potential implications for understanding the coordinated roles of multiple genes on cell signaling pathways^42^. A number of gene pairs with particular combinatorial mutational patterns have been identified by statistical analysis^28^, but this work was based on cell line data with very few samples. The current COSMIC database includes many clinical/genome-wide screened tumor samples for each cancer type, facilitating a more complete investigation of combinatorial mutational patterns. By calculating the likelihood ratio and statistical significance of gene pairs as co-mutational or mutually exclusive patterns, we tested a list of gene pairs with mutation frequency above certain threshold for each cancer type (Methods). Figure 7 illustrates the significant gene pairs with mutually exclusive patterns for large intestine, lung, and endometrial cancers. Figure S34 shows the other 9 cancers for which the statistical analysis identified at least two exclusive gene pairs. Gene pairs were saved in a text file, with each row corresponding to an exclusive gene pair, and input to Cytoscape^43^ (v3.0.2) to plot the final network (Fig. 7); colors and shapes were manipulated for better visualization. A complete list of significant gene pairs of co-mutational patterns for each cancer type is given in Table S3. Our study achieved a much greater coverage of relevant gene pairs across most cancer types compared to earlier work. Furthermore, our results are expected to be more reliable since 1) the mutation data employed were from clinical samples instead of cell lines data, and 2) our significance control for the co-occurrence pattern is more reasonable and more rigorous than previously used (Methods).

The gene pairs with significant exclusive patterns further verified the previous assumption that genes functioning in the same pathway are likely to mutate in an exclusive manner. For example, KRAS and BRAF gene mutations exclusively exist in colorectal cancers^44^, whereas EGFR mutations rarely co-occurred with KRAS in any cancer type^45^. Such functionally linked gene pairs were largely identified as exclusive patterns in our screening (Fig. 7). Remarkably, the APC and CTNNB1 (NH2-terminal domain) gene mutations were previously reported as mutually exclusive in colorectal cancers^46^, assuming both genes function in the APC/β-catenin/Tcf pathway. However, we found that these two genes cannot be categorized into any combinatorial pattern in large intestine cancers. Actually, among 599 genome-wide screened large intestine cancer samples, CTNNB1 (encoding β-catenin protein) was mutated in 99 samples, APC was mutated in 427 samples, and both genes were mutated in 78 samples. The likelihood ratio *LR* = 1.1052 is significantly smaller than the lower bound of the thresholds (Table S3), which means it should be an exclusive pattern (Methods). However, our calculation showed it was not statistically significant (P  >  0.5). The mutation rate of APC in large intestine cancers is much higher than that of CTNNB1, and the samples harboring APC mutations contained most of those harboring CTNNB1 mutations (78 of 99). Hence, this pattern is very different from the exclusive one and cannot be categorized into a mutually exclusive pattern. In fact, some researchers have referred to this type of pattern as a subsumed relation^28^. Here, the subsumed relation refers to the conjecture that the APC mutations probably precede the CTNNB1 mutations during carcinogenesis in the large intestine. The issue of temporal order (timing) of mutational events is discussed later.

The present study also demonstrated heterogeneity in combinatorial mutational patterns between cancer types. For instance, the KRAS gene mutated exclusively with the PTEN, VHL, RB1, and EGFR genes in large intestine cancers with high statistical significance. However, in lung cancers, KRAS mutated most frequently exclusive with the TP53, PKHD1, and SYNE1 genes. The KRAS gene also mutated exclusively with EGFR in lung cancers (Fig. 7 and Table S3). The gatekeeper gene TP53 was exclusive with different genes in almost all cancer types, albeit it generally maintains high mutation rate in those cancers (Fig. 3, Figure S34, and Table S3). This implies that the same gene may take part in various signaling pathways in different cancers, as revealed by the previous studies^1^.

The biological significance of co-mutational patterns, especially those that tend to simultaneously appear in different cancer types, deserves further experimental evaluation. We identified a batch of co-mutational gene pairs for various cancer tissues with high statistical significance (Table S3). These gene pairs were distinct across cancer types in general, but some simultaneously occurred in different cancers, e.g., NFATC4/FAT1 appeared in both endometrial and lung cancers and PEG3/ZIM2 appeared in skin and esophageal cancers. Since co-mutational genes are likely to function in distinct signaling pathways and exert joint effects on tumor progression, multiple oncogenic pathways driving tumor progression could be revealed by analyzing these co-mutational patterns. These considerations could be taken into account when designing drug combinations to target multiple signaling pathways simultaneously.

## Discussion

A comprehensive study of human cancer-specific mutational spectra is an important initial step towards distinguishing mutational patterns between cancers, identifying the most relevant cancer genes driving the tumorigenesis of particular cancer types, and elucidating etiological connections between specific mutagens and tumor evolution. The increasingly integrative COSMIC database, which incorporates considerable mutation records of clinical samples in the protein-coding genes, provides a unique opportunity for such comprehensive analysis. By analyzing more than one million missense mutations (involving ~8000 genome-wide screened samples across 23 major human cancers), we detected significant cancer-specific heterogeneity in several aspects, including the prevalence of point mutations, frequently mutated genes and mutational landscapes at the amino acid level, and combinatorial mutational patterns of related gene pairs. Compared to previous studies considering only nucleotide changes, our comprehensive investigation of amino acid substitutions and associated cancer genes revealed more significant cancer-specific heterogeneity, and hence allowed many novel insights into molecular mechanisms of tumor progression of particular cancer types.

Our mutation prevalence analysis revealed that solid tumors (especially self-renewing tissues such as colon and lung cancers) typically possess more mutations and more mutated genes than non-solid tumors (e.g., haematopoietic and lymphoid tissue cancers). For instance, an average colon tumor has 150 missense (a type of non-synonymous) mutations in protein-coding genes, while a hematopoietic or soft tissue cancer typically contains less than ten. One possible explanation is that liquid tumors do not need the extra mutations which enable them to metastasize, a key characteristic of malignant tumors^1^. However, skin cancers bear a median of only six missense mutations detected in the current COSMIC, much less than other typical solid tumors (Fig. 1). A quarter of the skin cancer samples maintain more than 125 mutations; yet over 25% of samples have only one or two mutations. The reason for this marked difference is unknown. Mutational frequency may vary widely between different subtypes of skin cancer, which needs further investigation. Another concern is the small sample size for some cancer types (e.g. adrenal gland, eye, and small intestine cancers), for which there are only 20 ~ 40 genome-wide screened samples in the current COSMIC database. A more integrative database containing sufficient cancer-specific mutations is essential for an unbiased analysis.

Cancer is widely considered as an age-associated disease - it needs time to accumulate the necessary somatic mutations that progressively change a selected clone from benign to malignant tumor^6^. This trend may also be detected through mutation occurrences across diagnosis age. Some cancers displayed strong correlation between patient age at diagnosis and mutation occurrences, e.g., esophageal, prostate, and stomach cancers. Older patients with these cancers tend to accumulate more somatic mutations. Other cancers exemplified no obvious age-mutation association, due to many possible reasons. In the current COSMIC release, there were no mutation records of patients aged 25 years or younger for self-renewing cancers such as colon and lung cancers; whereas, considerable numbers of patients under 25 years old were included in samples of other particular cancers, such as hematopoietic-lymphoid tissue and central nervous system tumors. However, this trend may not hold with more data coming into the database. In addition, since only a small fraction of samples has age information in the current COSMIC database, the correlation identified by the present study should be interpreted with caution due to its potential bias.

Since mutation information in COSMIC is manually curated from the scientific literature with precise definitions of disease types and patient details^47^, all mutation records contained in COSMIC are presumably associated with oncogenic progression to some extent. However, mutations on the highly mutated TTN and MUC16 genes were suspected of being neutral (passenger) mutations according to recent research^14^, and the potential biological mechanisms have been elucidated^19^. The evidence suggested that the high mutation frequency of olfactory receptor genes and some large genes (e.g. TTN and MUC16) could be attributed to their low expression level and late replication timing during the cell cycle. Our spectra analysis at the amino acid level identified distinct mutational spectra compared to other recognized cancer genes, suggesting their functional neutrality. However, considering their persistent presence in different cancer types (Fig. 3 and Table S2), and significant combinatorial mutational patterns (TTN tended to mutate exclusively with other genes, while MUC16 was likely to be co-mutational with others) (Fig. 7 and Table S3), we suggest that their role in cancer progression still remains to be evaluated. It would be interesting to distinguish cancer-associated genes from neutral ones based on our mutational spectra study at the amino acid level, but that question is not the focus of the current work.

The combinatorial mutational patterns of gene pairs (co-mutational versus exclusive patterns) have many ramifications in inferring signaling network modules for specific cancer types. Our investigation has identified considerable numbers of candidate gene pairs with significant biological relevance. Some results recapitulated previous observations, while others deserved further experimental validation. Besides the combinatorial mutational patterns, these cross-sectional data may also contain information related to the temporal order of two mutational events^28^,^48^, such as the aforementioned APC and CTNNB1 mutations. The temporal order of mutations is associated with stages of cancer progression^49^. Future studies will examine possible associations between the mutation frequency/sample coverage and the temporal order of gene mutations based on the integrative database.

## Methods

### Datasets and quality control

The current Catalog of Somatic Mutations in Cancer (COSMIC v68) contains 27 keywords to describe mutation and sample information, including the gene name and its alias ID in different data sources, the sample name/ID and source, the mutation detail in gene and its associated protein sequence, and whether it was genome-wide screened, etc. This version also contains patient age information for some samples.

The COSMIC v68 contains a total of 1,627,583 mutation records involving 235,589 samples. By extracting the column of keyword ‘Primary site’, we obtained 42 major human cancer types (differing in tissue types) plus some mutations of non-specific tissue origin (denoted ‘NS’), which can be further categorized into 190 subtypes according to ‘Site subtype’. These mutations involved ~20,000 human genes in total with heterogeneous coverage over different cancer types (supplementary Table S1).

In the present study, we only considered the genome-wide screened samples and excluded synonymous mutations denoted as ‘coding silent’. The first criterion (genome-wide screening) filtered out about 16% of the original records, and the second principle (non-synonymous) further excluded 21% of the remaining ones. Some cancers (e.g. pleura, pituitary, and testis) did not meet both criteria and were excluded from later analysis. We obtained 28 cancer types through this initial screening (excluding ‘NS’). The sample size for some cancer types are below 20, which is too small for statistical analysis. These cancer types were also removed, including thyroid, soft tissue, cervix, biliary tract, and parathyroid cancers. Hence, our analysis included the remaining 23 cancers (supplementary Table S1). Here the threshold of 20 samples was chosen to satisfy two primary goals: first, each cancer type under investigation has a reasonably large sample size, to minimize statistical bias; second, our investigation could support a meaningful comparative study across various cancer types. Mutation records were first extracted separately based on different tissue types. After that, for each cancer type, mutations together with other information were grouped sample by sample, and mutated genes of each sample were collected for further analysis.

### Mutational analysis at the amino acid level

The amino acid substitutions were extracted from the mutation records under the key ‘Mutation AA’ and denoted as a 2-gram code for the amino acid mutation and a positive integer number for the mutation position. For example, the mutation record ‘p.A593E’ contains the 2-gram code ‘AE’ and the position 593. For the 20 amino acids {‘ACDEFGHIKLMNPQRSTVWY’}, there are 380 combinations of any two distinct characters, corresponding to 380 different amino acid substitutions. For each cancer type, we first generated all the 2-gram codes for the amino acid mutations, and then calculated the frequency of mutations along the 380 residue alterations. After that, alterations that were extremely rare across cancer types were removed since these substitutions contribute little to discriminating between molecular subtypes. In our practice, if sum of the frequency of occurrence across all cancers was lower than 1% for an amino acid substitution, it was excluded. This procedure yielded 149 significant amino acid substitutions. Based on their frequency distribution along these 149 amino acid substitutions, cancers were clustered (average-linkage, Euclid distance) into several groups.

### K-S test for mutational preference across chromosomes

We employed the Kolmogorov-Smirnov test (K-S test) to determine whether somatic mutations for a cancer type occur preferentially in particular chromosomes. The K-S test statistic quantifies a distance between the empirical distribution functions of the test sample and that of the reference sample to determine whether the test sample is drawn from the reference distribution. In the present study, we took chromosome lengths as the reference sample. Our purpose was to see whether there are significantly more somatic mutations on the longer chromosomes for each cancer. To achieve this, we first calculated the cumulative length proportion of each chromosome among the whole genome. Then we determined the number of mutations in each chromosome and determined their cumulative probability of occurring in each chromosome. We denote the cumulative distribution function of the reference and test sample as *F*_*r*_*(x)* and *F*_*t*_*(x)*, and then the K-S statistic can be represented as equation (1).
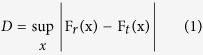
This statistic reflects the difference between the reference and test distribution. In our study, distributions with *D* > 0.05 were considered as significantly different (mutations of that cancer exhibit certain chromosome preference).

### Likelihood ratios of combinatorial mutational patterns and statistical significance

Previous experimental and statistical studies have consistently identified two combinatorial mutational patterns for gene pairs in a tumor sample, termed co-mutational and mutually exclusive patterns^1^,^15^. The co-mutational pattern occurs when two genes tend to mutate simultaneously in a single tumor, while the mutually exclusive pattern occurs when one and only one of a pair of genes mutates in any single tumor. Mutually exclusive genes may tend to function in the same signaling pathway, while co-mutational genes may be likely to take effect in different pathways^30^. Hence, identifying gene pairs with obvious combinatorial mutational patterns has significant biological meaning.

To determine combinatorial mutational patterns, we first determined the candidate gene pairs both mutated in at least 10%, 5% and 2% of the dominancy, average, and non-dominancy cancer samples, respectively (Fig. 3). Then we calculated a likelihood ratio (*LR*_comb_) between the empirical co-occurrence frequency and the expected co-occurrence frequency according to the simplest model^28^. The ratio can be mathematically expressed as equation (2).
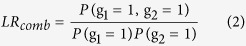
Where P(g_i_ = 1) and P(g_1_ = 1, g_2_ = 1) stand for the probability that a single or both genes are mutated across samples, respectively. Note that for the exclusive pattern, the smaller the likelihood ratio, the more likely the related genes are mutated exclusively; while for the co-mutational pattern, the trend is opposite. To obtain cutoff value(s) distinguishing between co-mutational and exclusive patterns, we applied the mixture Gaussian distribution fitting model using Expectation-Maximization algorithm^50^. Suppose *m*_*1*_, *m*_*2*_ are the means of the low and high components, and *δ*_*1*_, *δ*_*2*_ their standard deviations. Then the thresholds for the co-mutational pattern (lower bound) and exclusive pattern (upper bound) are calculated as θ_1_ = m_2_−δ_2_/2 and θ_2_ = m_1_+δ_1_/2, respectively. In our study, this cutoff varied between cancer types (Table S3).

We measured the significance of the combinatorial mutational patterns of gene pairs by calculating the *p*-value of a hyper-geometric test. Suppose in a population of *n* samples, *n*_*1*_ carry a gene 1 mutation, *n*_*2*_ carry a gene 2 mutation, and *n*_*12*_ carry both mutations. The *p*-value for mutual exclusion and co-occurrence can be calculated by equations (3) and (4), respectively.
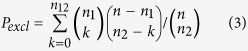

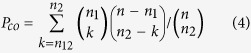
The difference lies in what events are considered as extreme cases in testing a statistical hypothesis. For exclusive patterns, we counted the number of times we observed at most n_*12*_ double mutations; for co-mutational patterns, we counted the times of observing at least *n*_*12*_ (up to *n*_*2*_) double mutations. We employed a different *p*-value formula for co-occurrence than previously shown in the literature^28^, since the previously used probability space does not sum to unity and consequently tends to produce smaller *p*-values than normal.

## Additional Information

**How to cite this article**: Tan, H. *et al.* Genome-wide mutational spectra analysis reveals significant cancer-specific heterogeneity. *Sci. Rep.* **5**, 12566; doi: 10.1038/srep12566 (2015).

## Supplementary Material

Supplementary Information

Supplementary Information

Supplementary Information

Supplementary Information

## Figures and Tables

**Table 1:** Top frequently occurring amino acid substitutions detected in COSMIC in comparison with prevalent nucleotide variations detected in TCGA.

Cancer tissue	**Top 3 amino acid substitutions (associated nucleotide variations)**			Prevalent nucleotide variations by Alexandrov *et al*.
	1st	2nd	3rd	
lung	GV(GT)	EK(GA)	RL(GT)	C > T, C > A
urinary_tract	EK(GA)	EQ(GC)	DN(GA)	C > T, C > G in bladder cancer
large_intestine	RH(GA)	RQ(GA)	RC(CT)	C > T in colorectum
esophagus	RH(GA)	RC(CT)	RQ(GA)	C > T, C > G
endometrium	RQ(GA)	RH(GA)	RC(CT)	C > T, C > G in cervix and uterus
liver	IV(AG)	AT(GA)	YC(AG)	C > T, C > A,T > C
stomach	RH(GA)	RQ(GA)	RC(CT)	C > T, C > G,T > C
kidney	AV(CT)	AT(GA)	RH(GA)	C > T, C > G
ovary	RH(GA)	AT(GA)	AV(CT)	C > T
breast	EK(GA)	EQ(GC)	RH(GA)	C > T, C > G
prostate	RH(GA)	RC(CT)	AT(GA)	C > T, C > A
upper_aerodigestive_tract	EK(GA)	DN(GA)	EQ(GC)	N/A
pancreas	RH(GA)	RC(CT)	AV(CT)	C > T, C > G,C > A
bone	RC(CT)	RH(GA)	VI(GA)	C > T, C > G in myeloma
eye	QL(AT)	AT(GA)	RC(CT)	C > T, C > A in head and neck
autonomic_ganglia	AS(GT)	QK(CA)	AT(GA)	N/A
salivary_gland	RH(GA)	RC(CT)	AT(GA)	C > T, C > A in head and neck
hematopoietic_and_lymphoid_tissue	RH(GA)	RC(CT)	AV(CT)	C > T, C > G,T > G in AML,ALL,CLL and lymphoma B cell
skin	EK(GA)	PS(CT)	SF(CT)	C > T in melanoma
central_nervous_system	RH(GA)	RQ(GA)	RC(CT)	C > T, C > A
meninges	KQ(AC)	RH(GA)	TI(CT)	N/A
adrenal_gland	GR(GA,GC)	LR(TG)	LV(CG,TG)	N/A
small_intestine	AV(CT)	RH(GA)	RQ(GA)	N/A

GV: amino acid residue G is mutated to V. Corresponding nucleotide changes inferred from the DNA codon table are given in parentheses. N/A = cancer type not covered by previous literature.
