# Supplementary material for: Genome-wide mutational spectra analysis reveals significant cancer-specific heterogeneity
Source: Sci Rep. 2015 Jul 27;5:12566. doi: 10.1038/srep12566 (PMC4515826; doi:10.1038/srep12566)
Supplement: Supplementary Information [file srep12566-s1.pdf]

Supporting Information for

Genome-wide mutational spectra analysis reveals significant cancer-specific heterogeneity

Hua Tan[1,3], Jiguang Bao[2], and Xiaobo Zhou[1,*]

[1]Center for Bioinformatics & Systems Biology, Department of Radiology, Wake Forest School of Medicine, Winston-Salem 27157, USA

[2]School of Mathematical Sciences, Beijing Normal University, Beijing 100875, China

[3]College of Global Change and Earth System Science, Beijing Normal University, Beijing 100875, China

Hua Tan warm.tan@gmail.com; htan@wakehealth.edu

Jiguang Bao jgbao@bnu.edu.cn

Xiaobo Zhou xizhou@wakehealth.edu

Summary of supporting information

This file includes:

Figure S1-S23: distribution of somatic mutations across the chromosomes for 23 major human cancers by ‘rainfall’ plots.

Figure S24: K-S test for mutant gene number distribution along the chromosomes.

Figure S25: mutation distribution along 380 amino acid substitutions.

Figure S26: mutation frequency averaged on 23 cancers and clustering results.

Figure S27: mutation distribution along 12 nucleotide base pair changes.

Figure S28-S33: mutational spectrum at the amino acid resolution for KRAS, PIK3CA, PTEN, APC, TTN and MUC16 genes respectively.

Figure S34: gene pairs with significant exclusive pattern detected in 9 cancer types.

Figure S35: protein sequence length relative to TTN for the top 1000 frequently mutated genes.

Statistical significance analysis on cancer-specific mutation frequency of genes

Statistical significance analysis on frequency of arginine (*R*) substitution

Other material as separate files:

Table S1: a summary of the COSMIC v68 and the data used in this study.

Table S2: top frequently mutated genes in general and across cancer types.

Table S3: gene pairs with significant combinatorial mutational patterns in various cancer types.

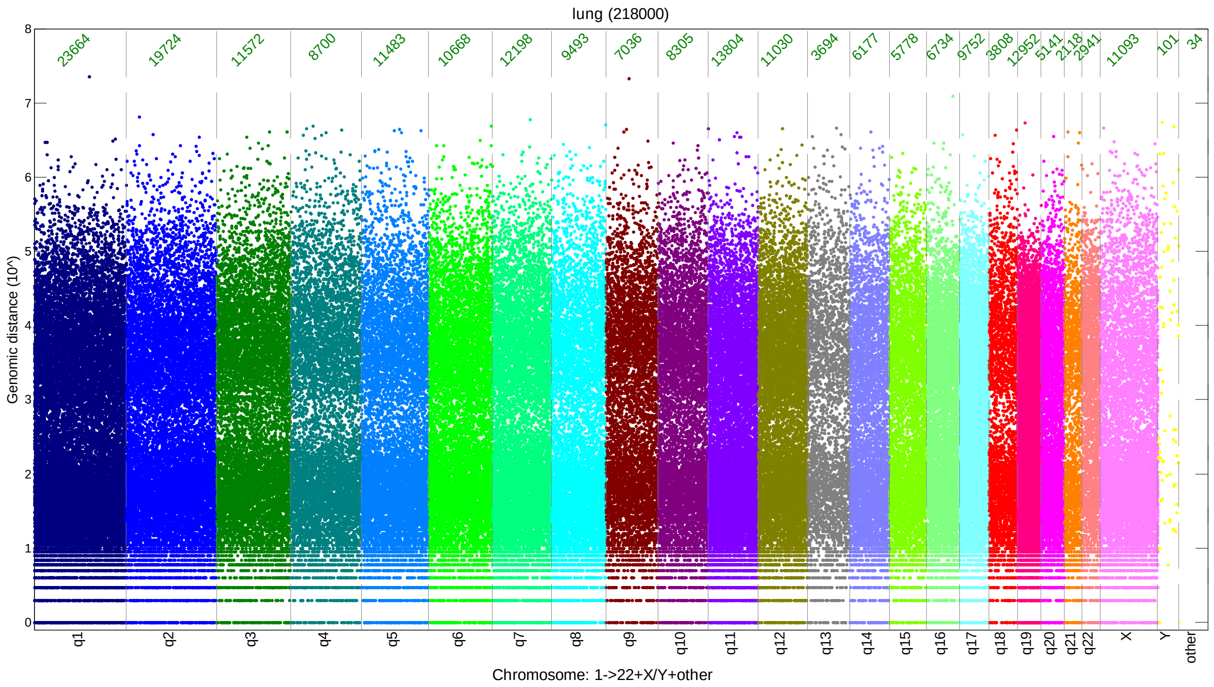

Figure S1 Mutation distribution across the chromosomes for 858 genome-wide screened lung tumor samples. Vertical axis denotes the genomic distance of each mutation from the previous mutation.

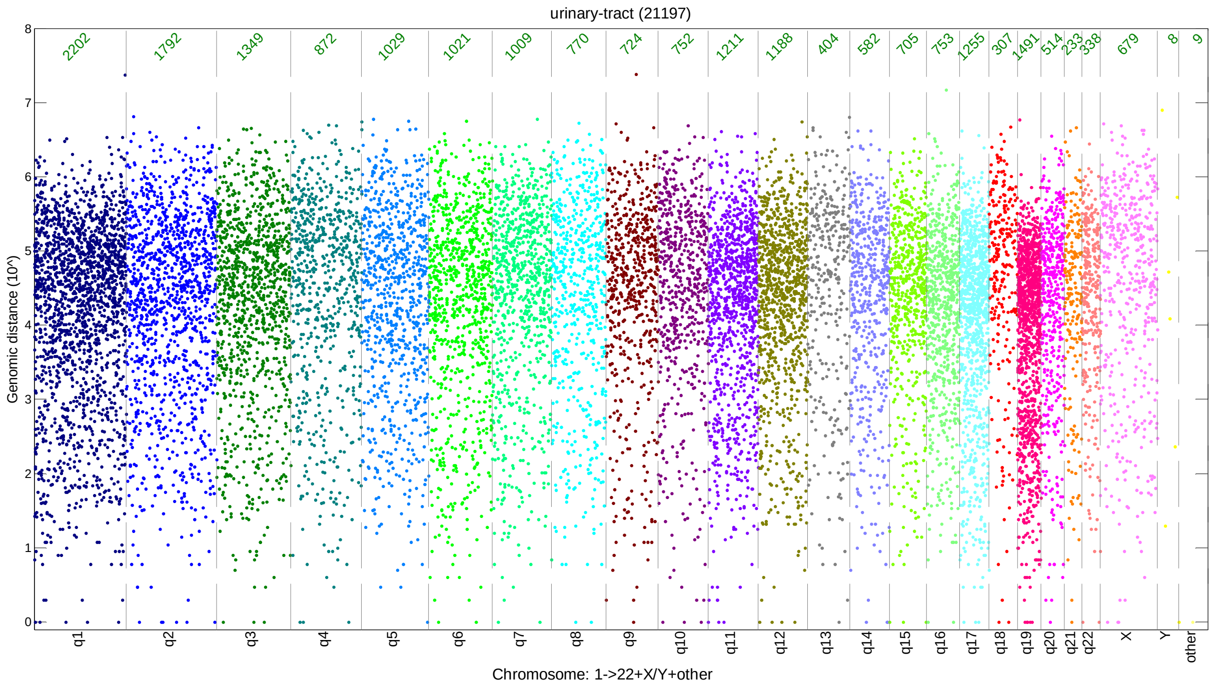

Figure S2 Mutation distribution across the chromosomes for 103 genome-wide screened urinary tract tumor samples.

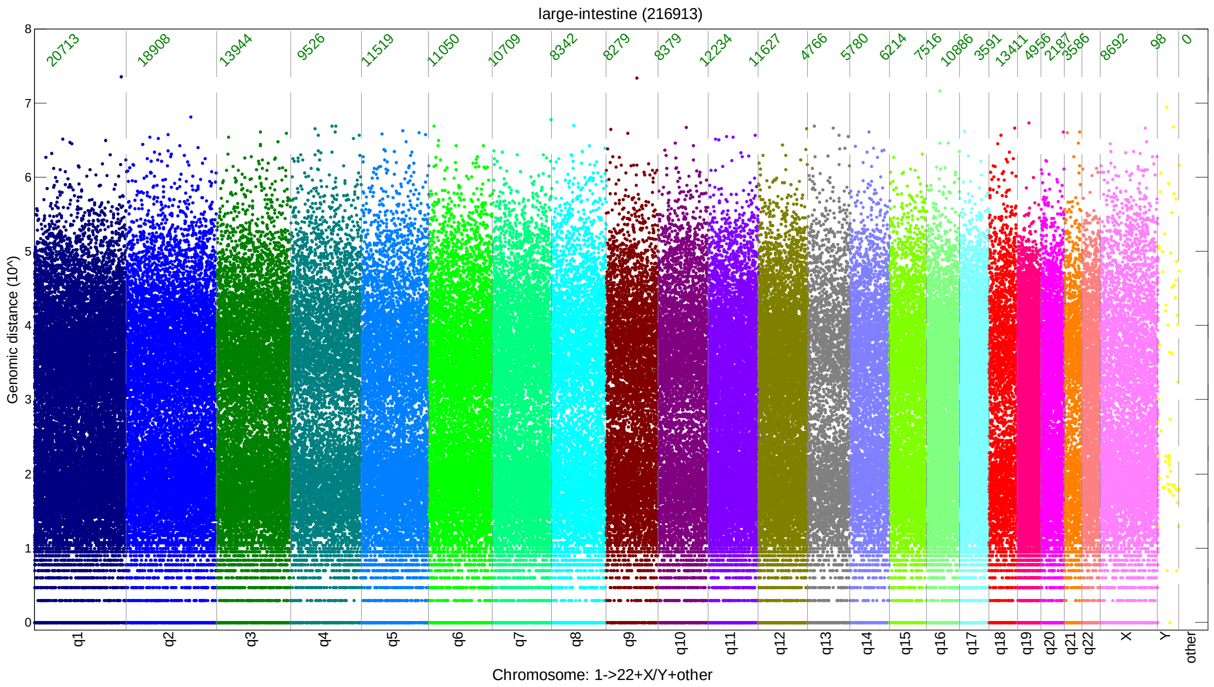

Figure S3 Mutation distribution across the chromosomes for 599 genome-wide screened large intestine tumor samples.

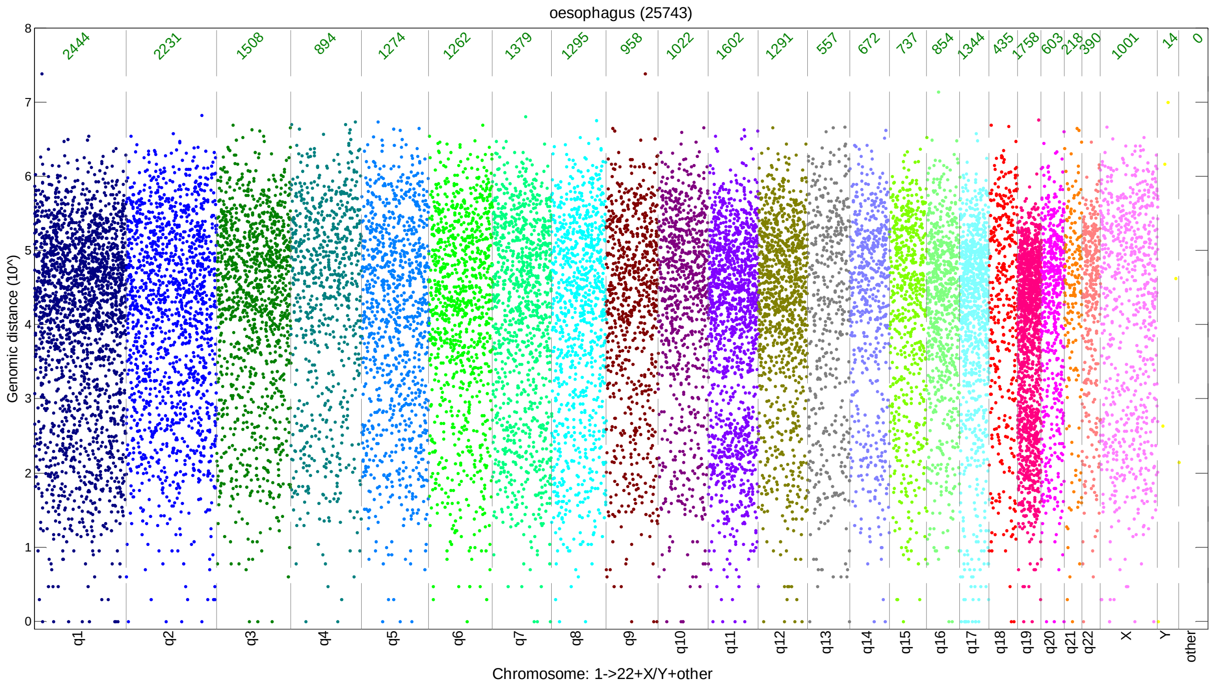

Figure S4 Mutation distribution across the chromosomes for 173 genome-wide screened oesophagus tumor samples.

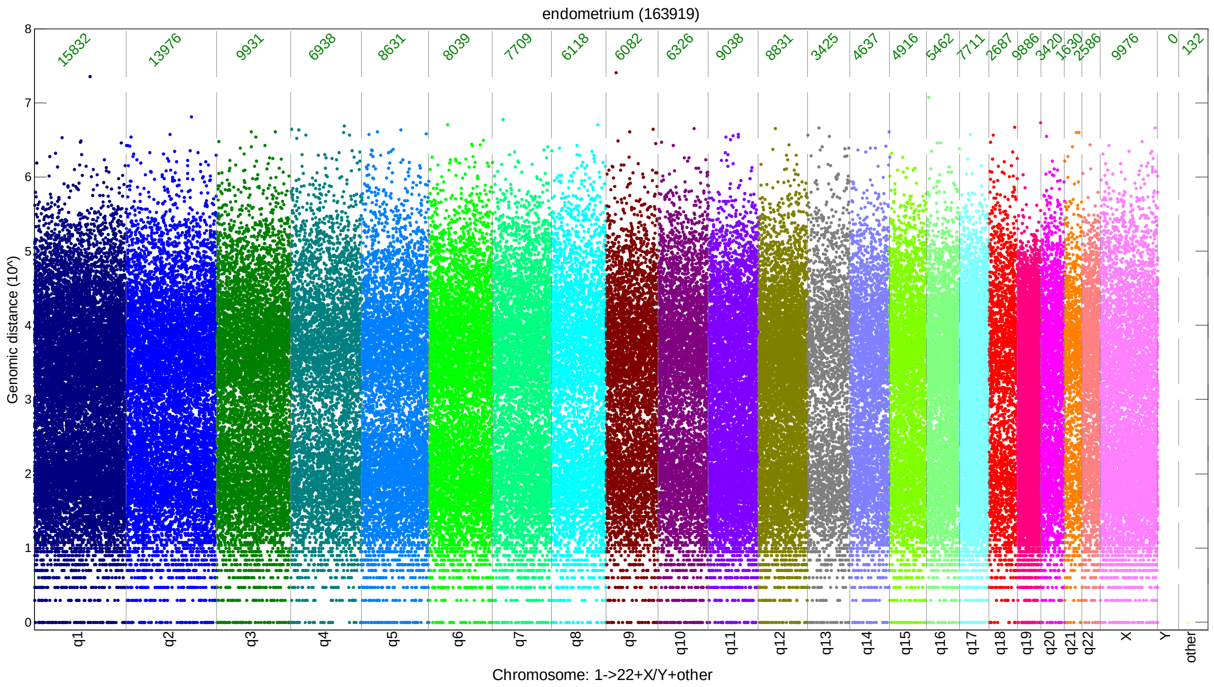

Figure S5 Mutation distribution across the chromosomes for 281 genome-wide screened endometrium tumor samples.

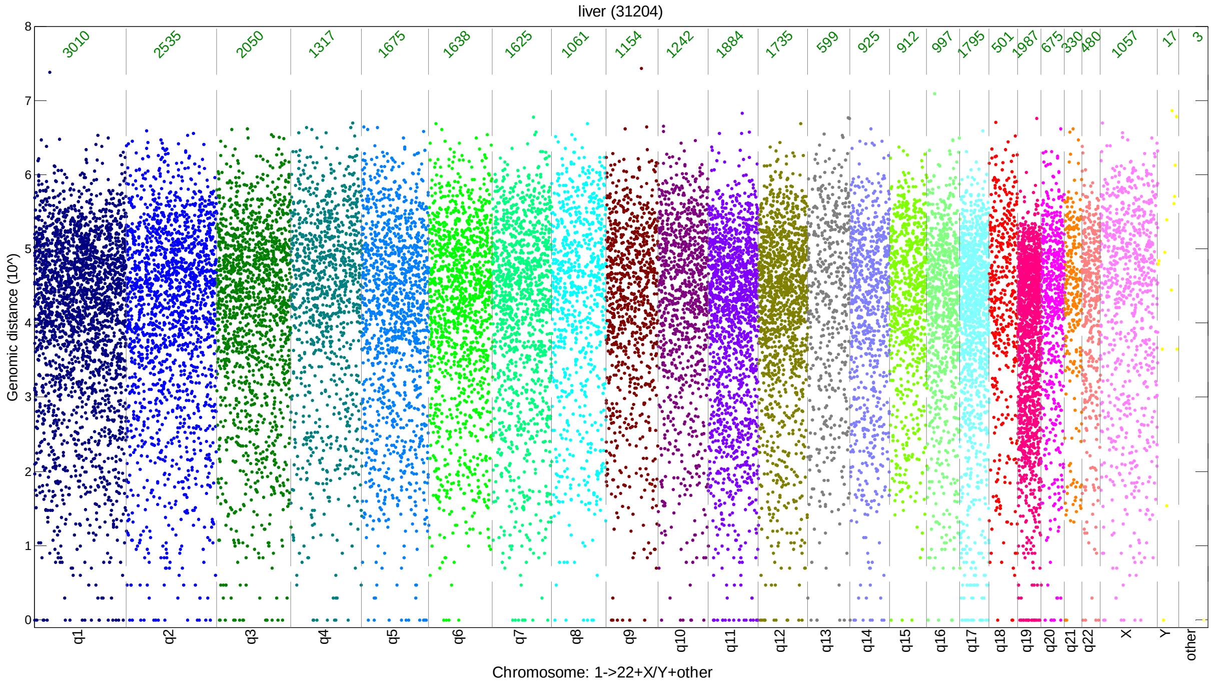

Figure S6 Mutation distribution across the chromosomes for 438 genome-wide screened liver tumor samples.

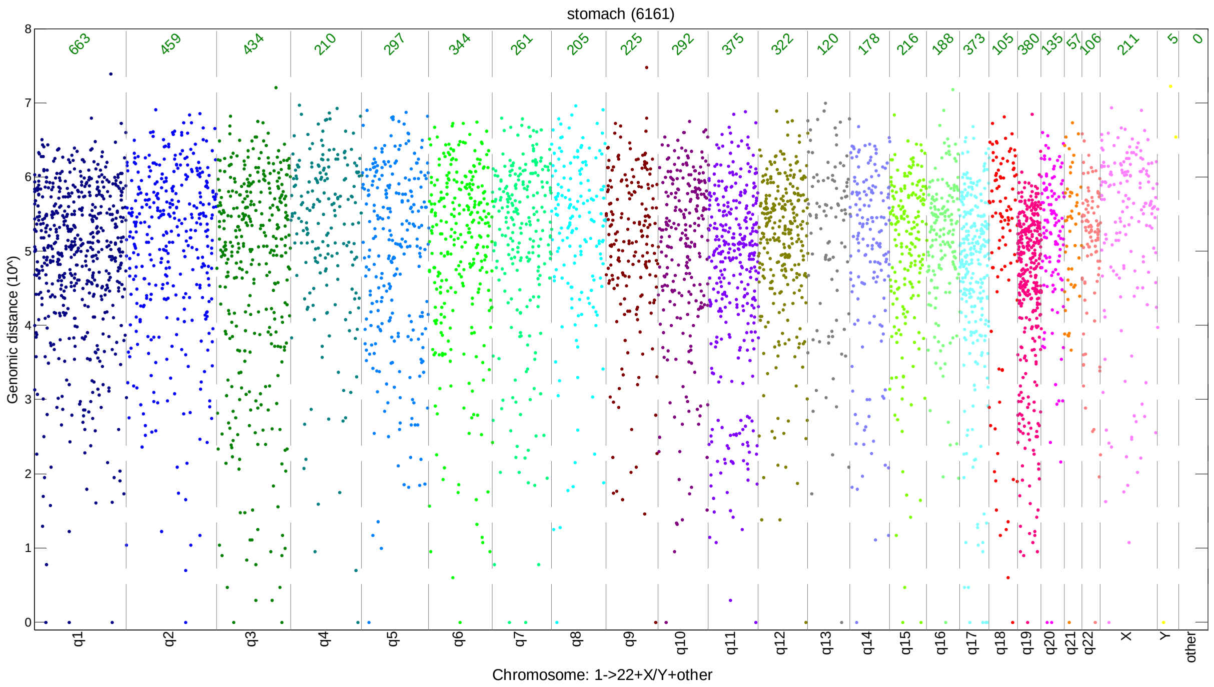

Figure S7 Mutation distribution across the chromosomes for 47 genome-wide screened stomach tumor samples.

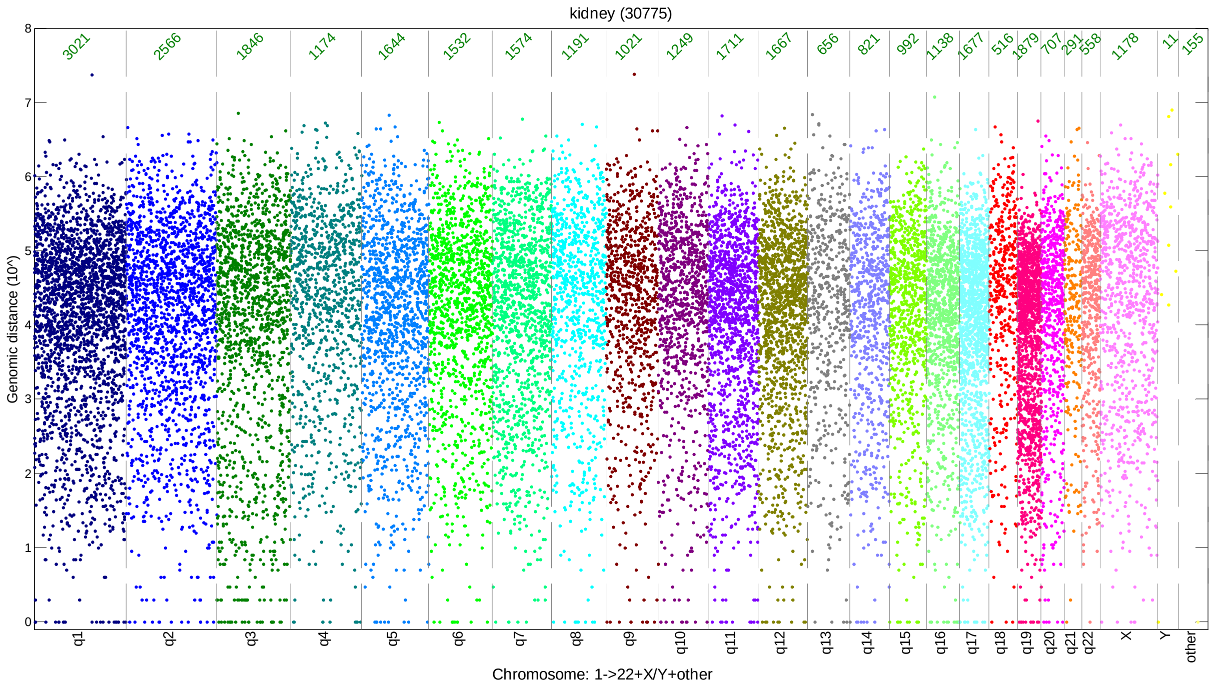

Figure S8 Mutation distribution across the chromosomes for 475 genome-wide screened kidney tumor samples.

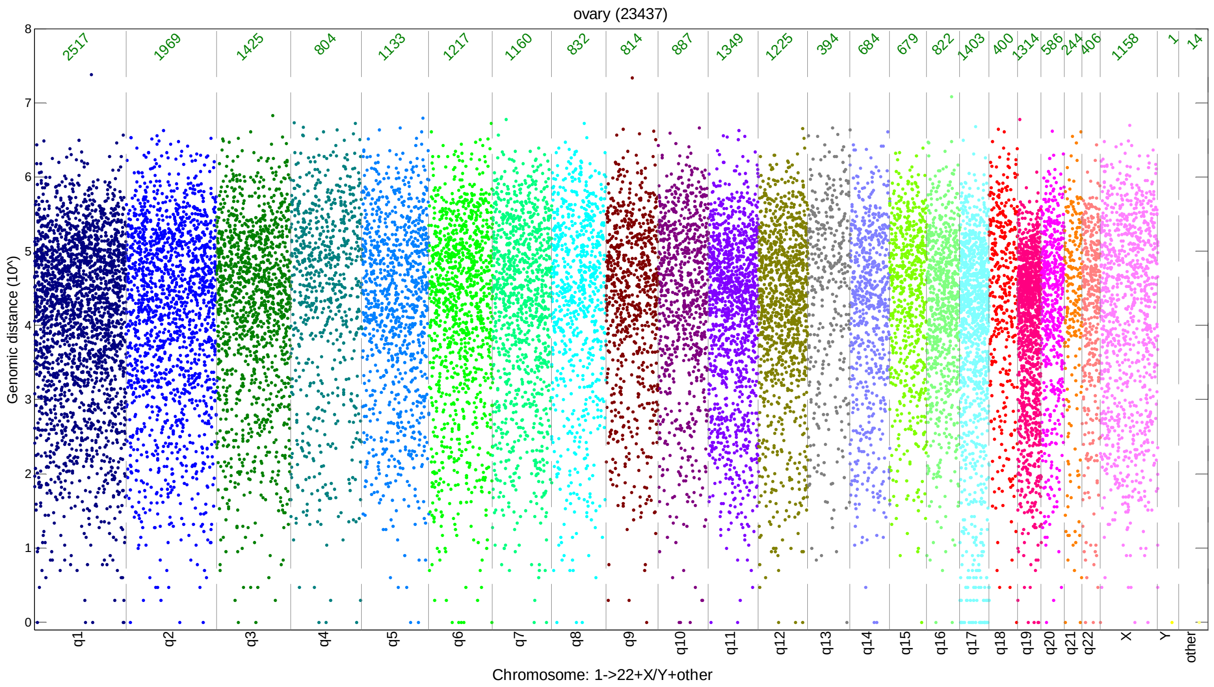

Figure S9 Mutation distribution across the chromosomes for 504 genome-wide screened ovary tumor samples.

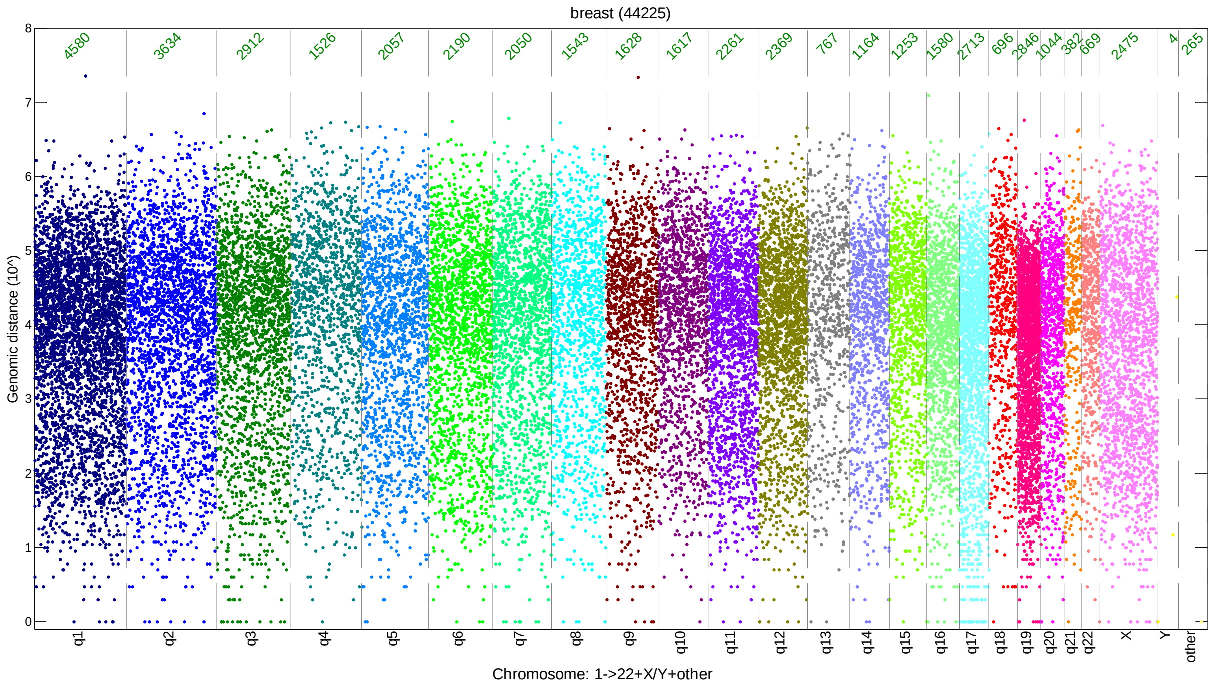

Figure S10 Mutation distribution across the chromosomes for 952 genome-wide screened breast tumor samples.

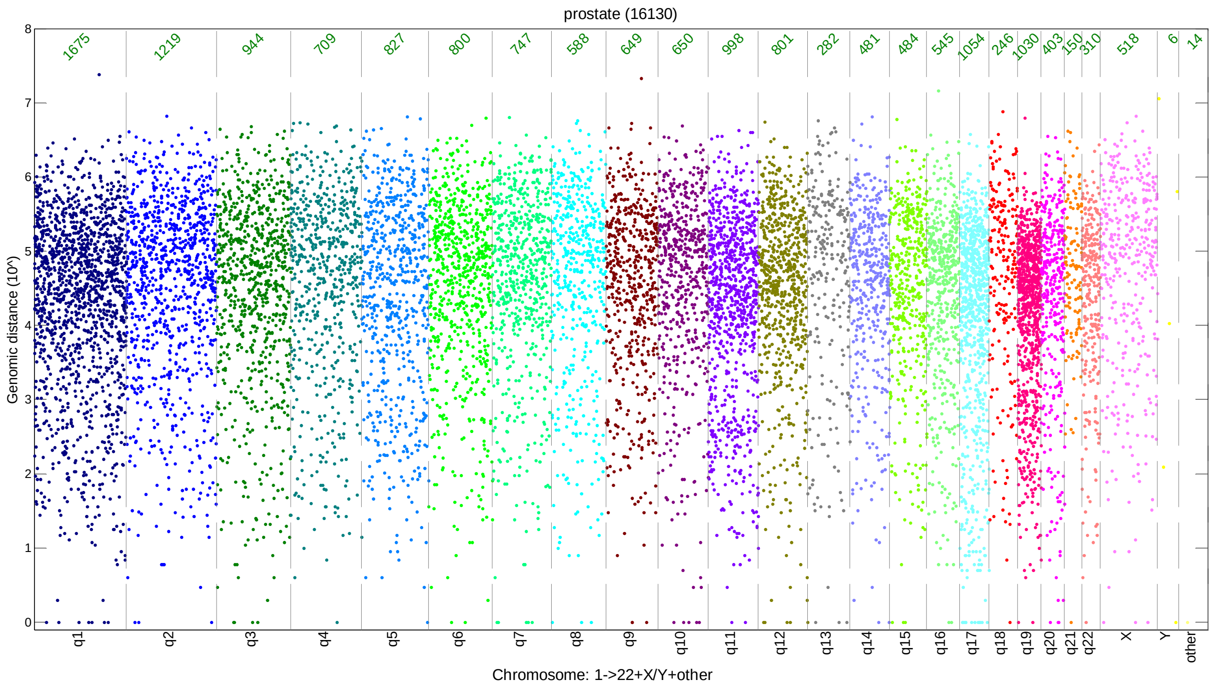

Figure S11 Mutation distribution across the chromosomes for 384 genome-wide screened prostate tumor samples.

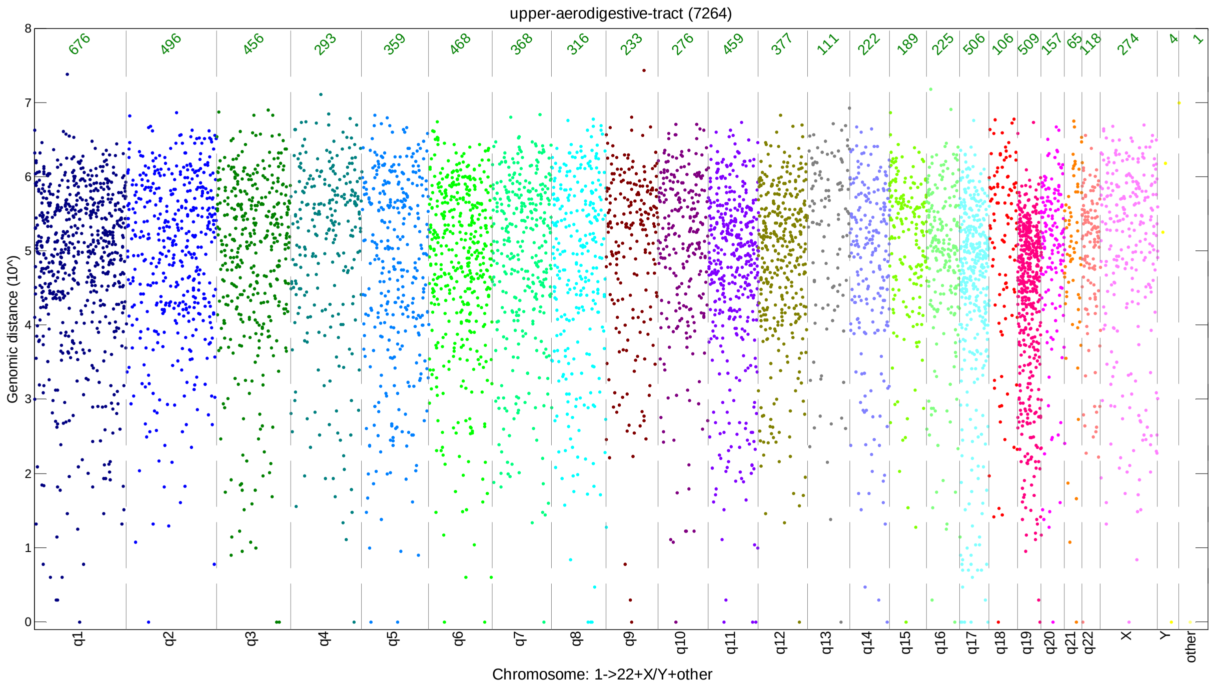

Figure S12 Mutation distribution across the chromosomes for 161 genome-wide screened upper aerodigestive tract tumor samples.

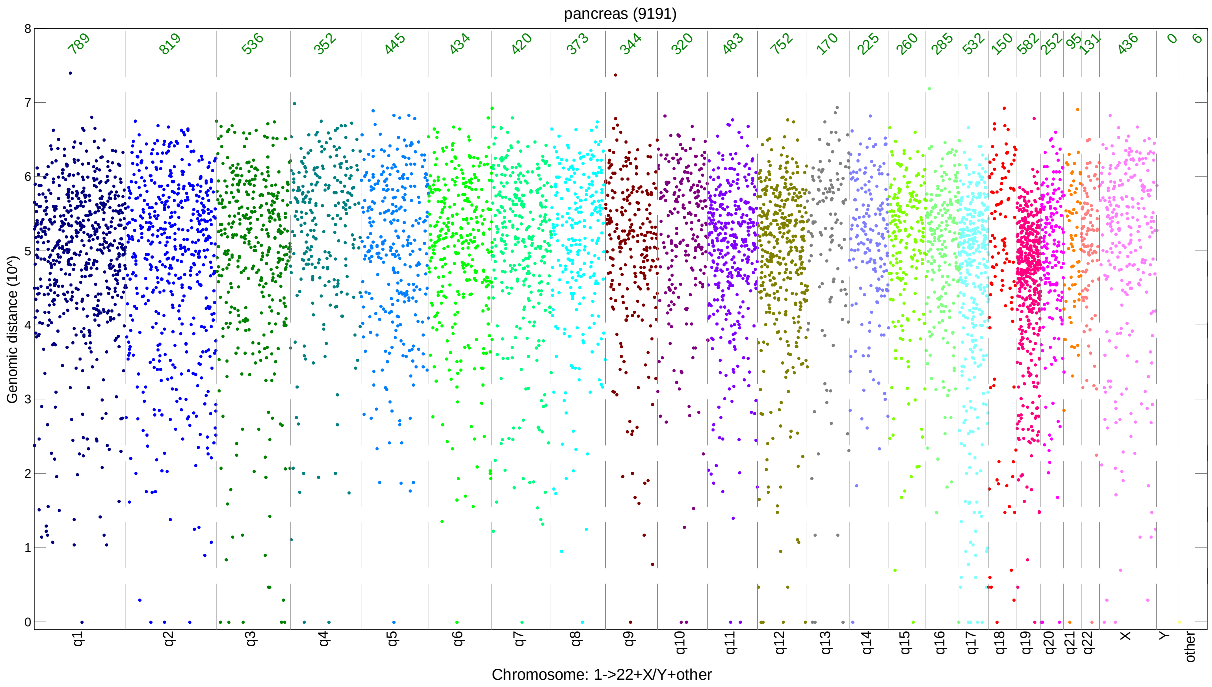

Figure S13 Mutation distribution across the chromosomes for 345 genome-wide screened pancreas tumor samples.

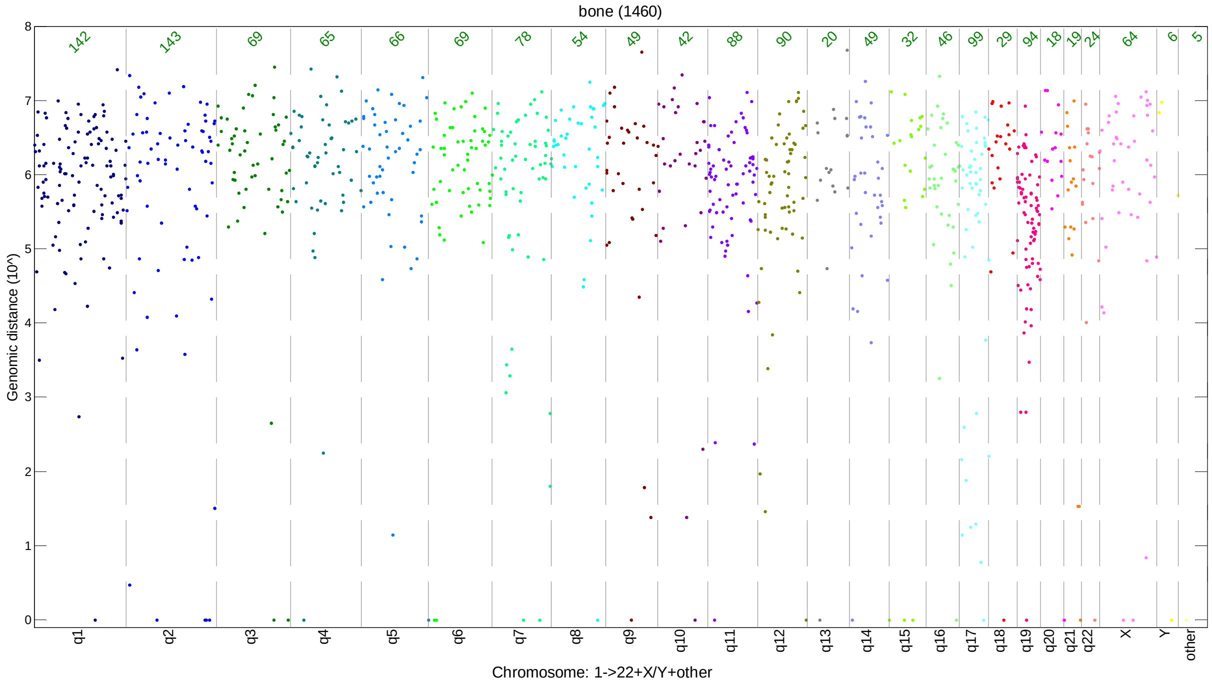

Figure S14 Mutation distribution across the chromosomes for 74 genome-wide screened bone tumor samples.

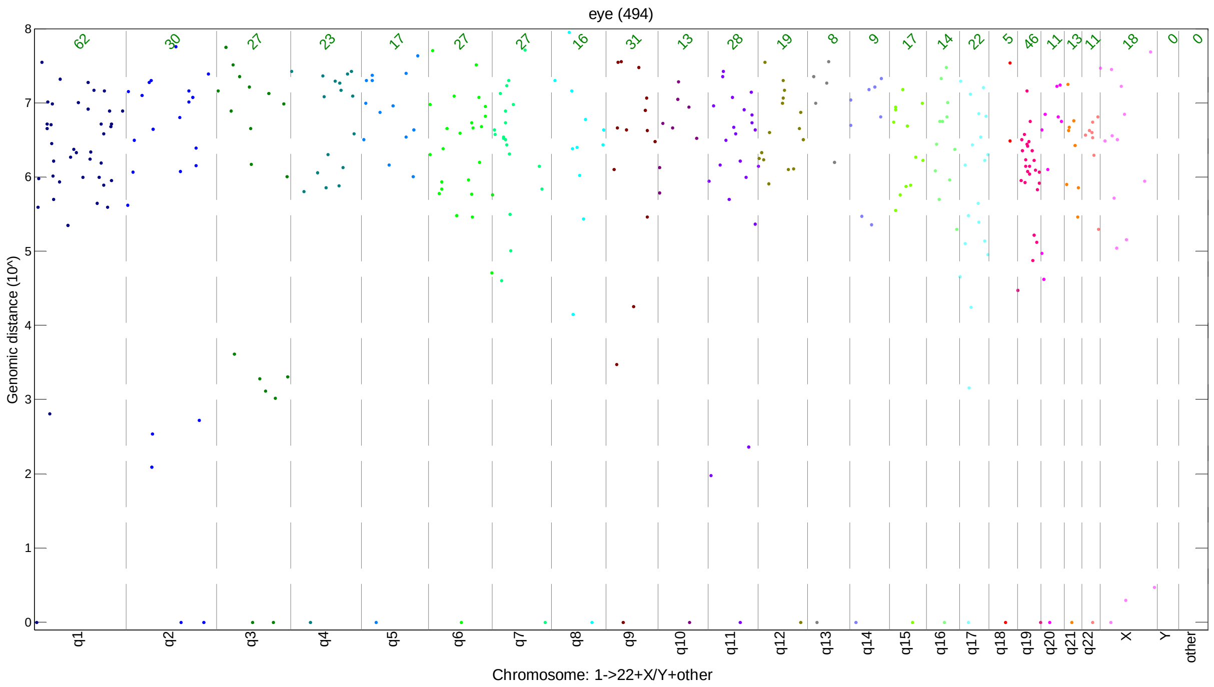

Figure S15 Mutation distribution across the chromosomes for 34 genome-wide screened eye tumor samples.

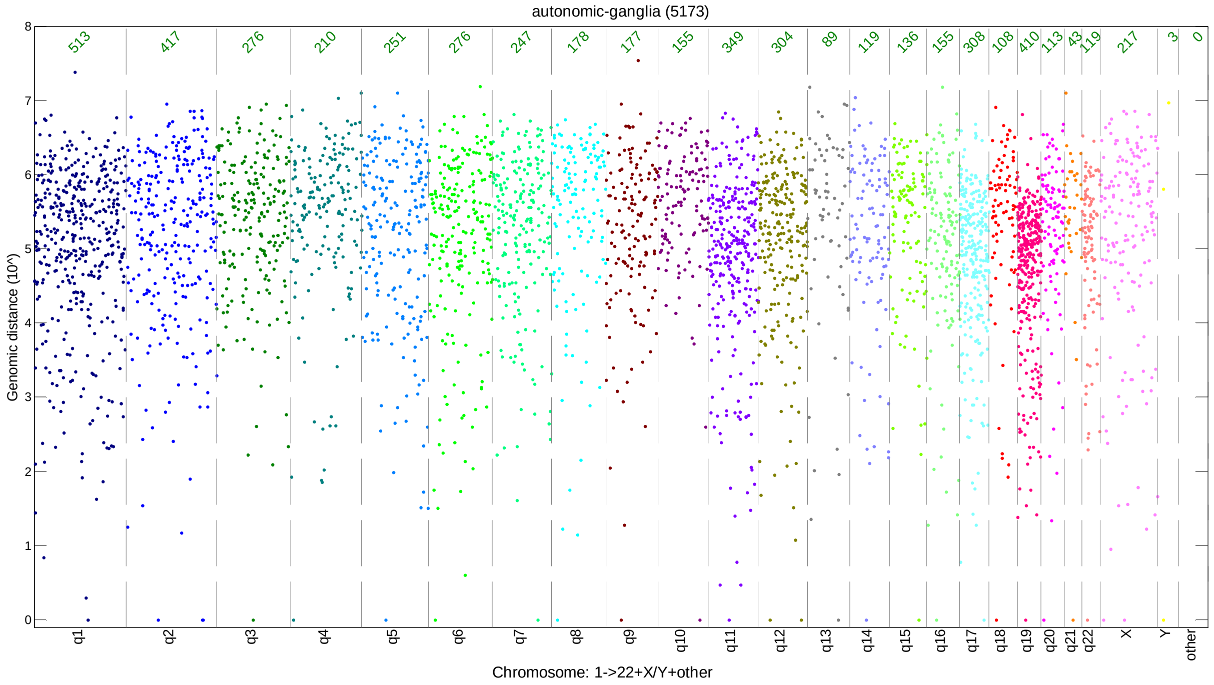

Figure S16 Mutation distribution across the chromosomes for 327 genome-wide screened automatic ganglia tumor samples.

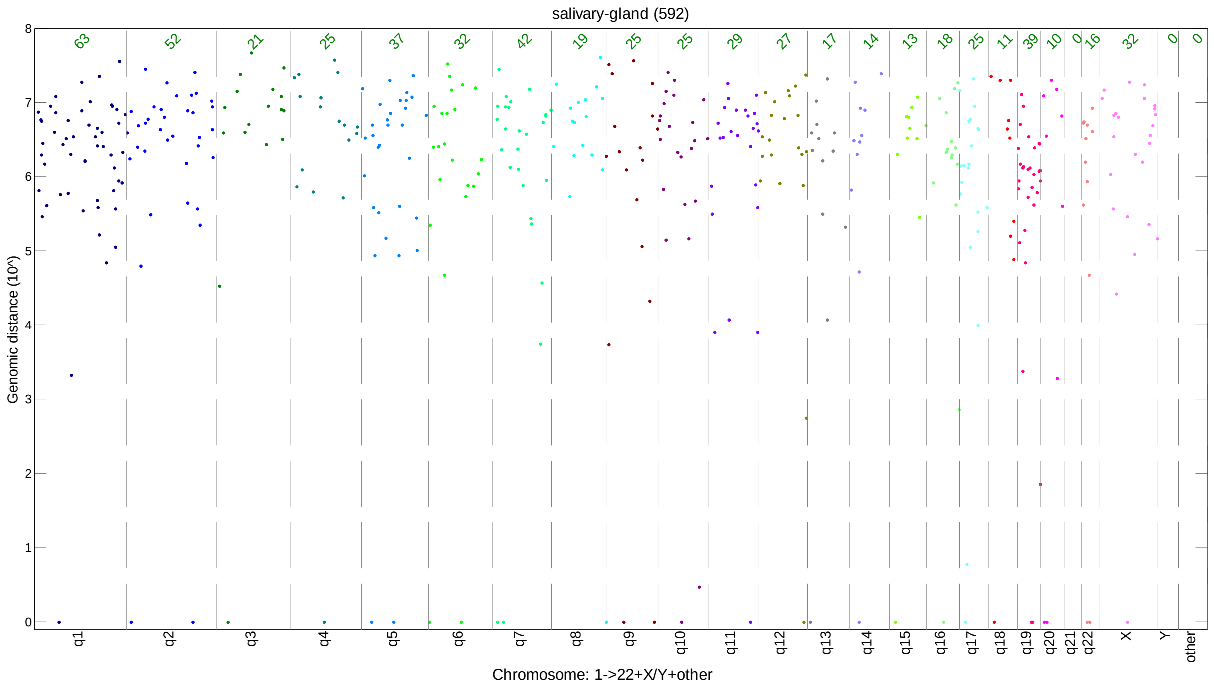

Figure S17 Mutation distribution across the chromosomes for 49 genome-wide screened salivary gland tumor samples.

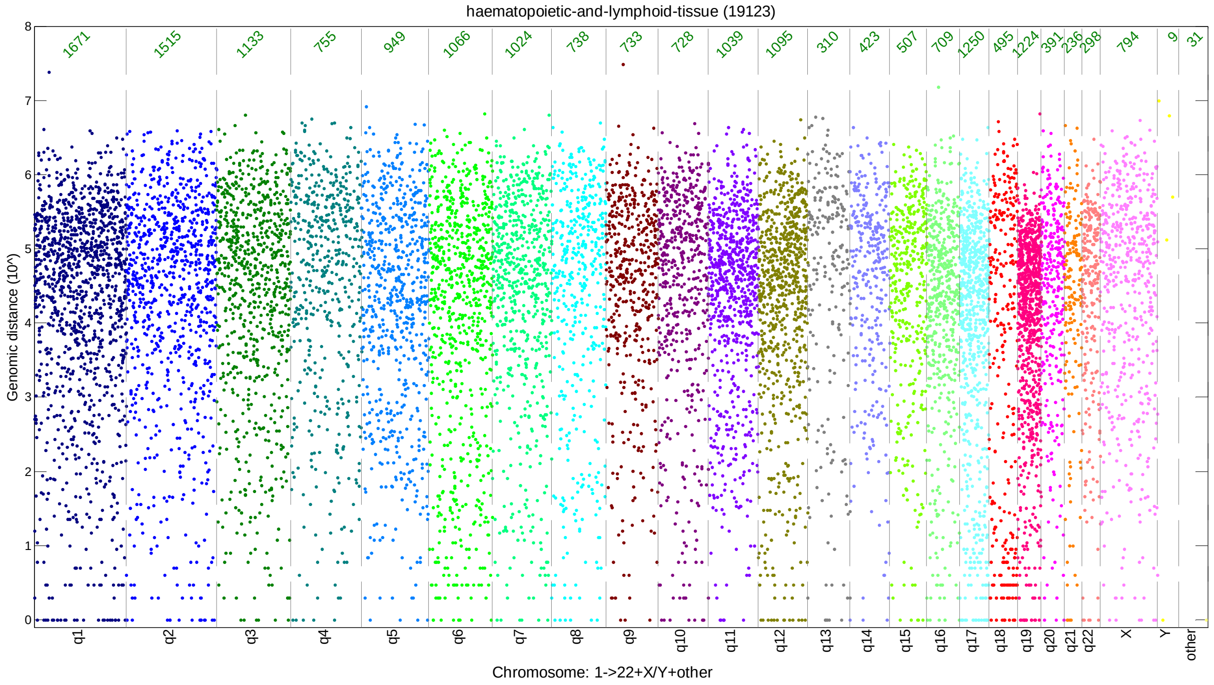

Figure S18 Mutation distribution across the chromosomes for 1008 genome-wide screened haematopoietic-and-lymphoid-tissue tumor samples.

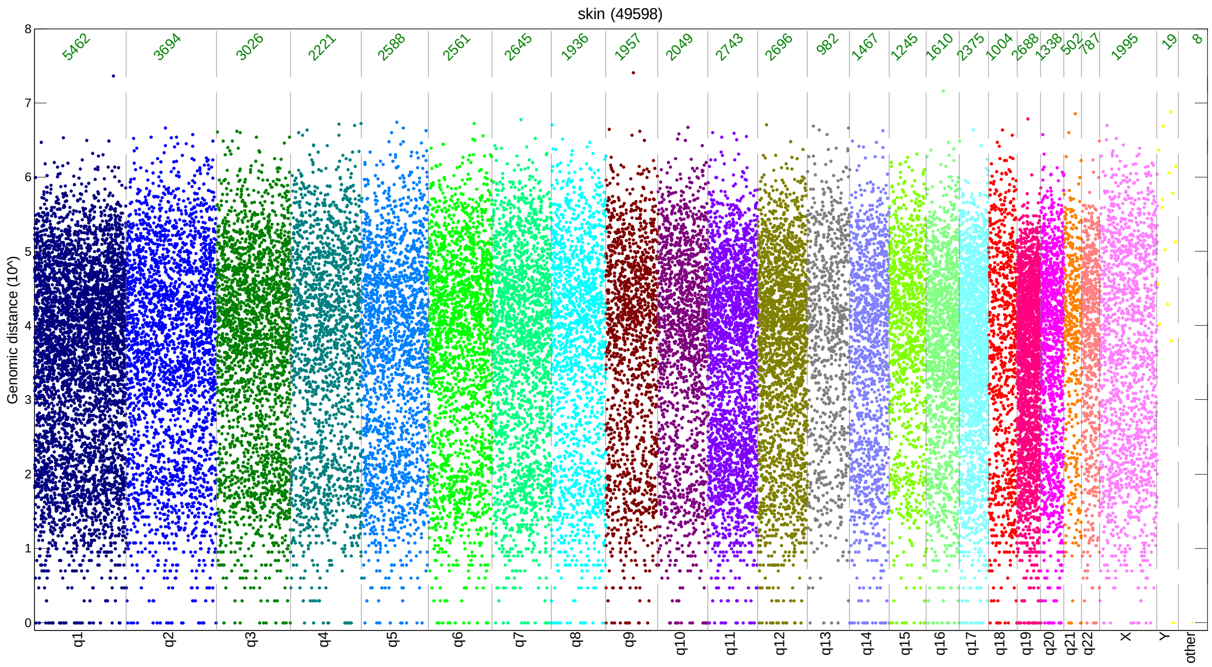

Figure S19 Mutation distribution across the chromosomes for 320 genome-wide screened skin tumor samples.

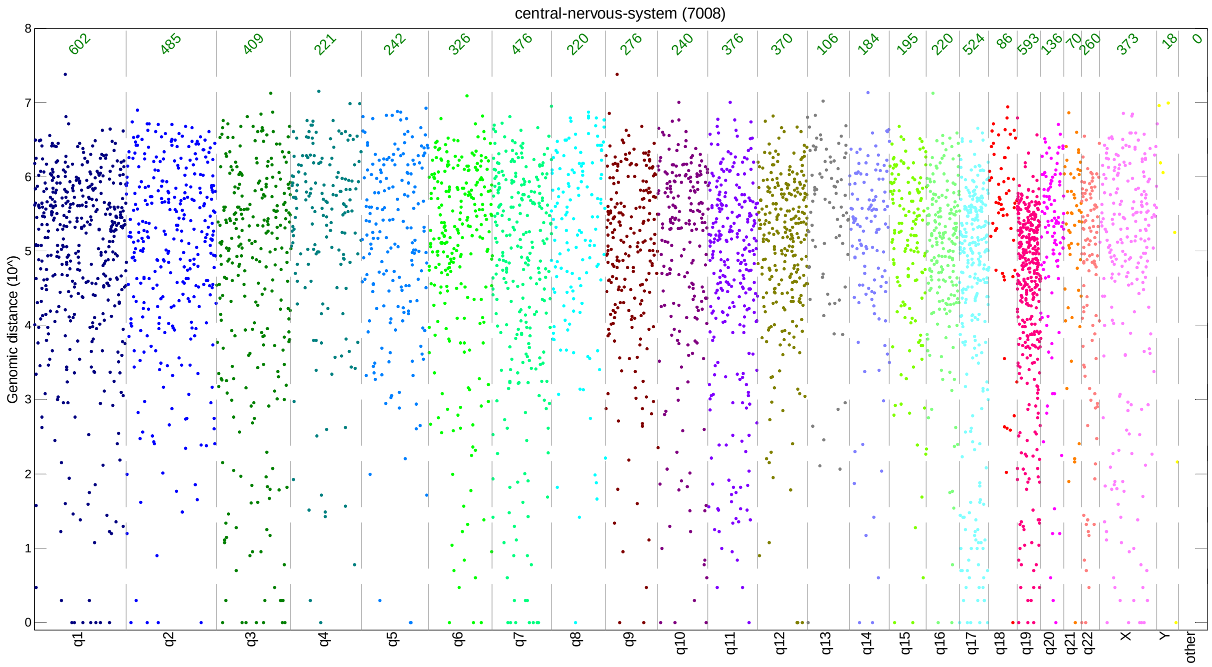

Figure S20 Mutation distribution across the chromosomes for 519 genome-wide screened central nervous system tumor samples.

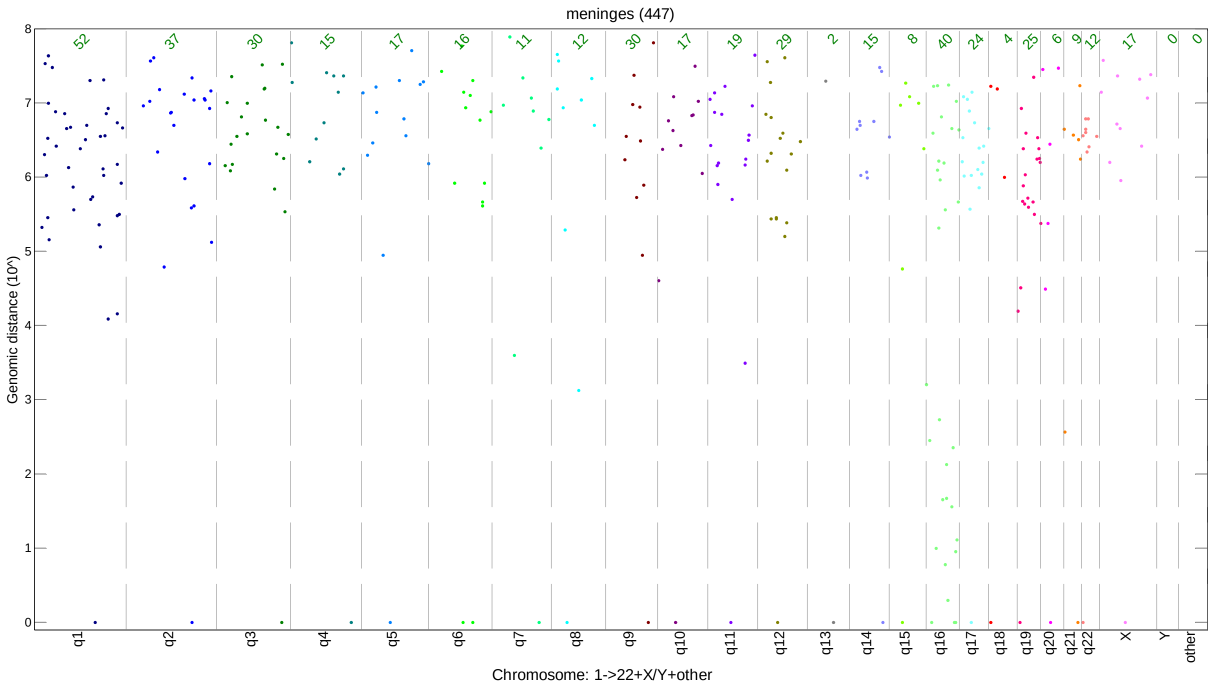

Figure S21 Mutation distribution across the chromosomes for 55 genome-wide screened meninges tumor samples.

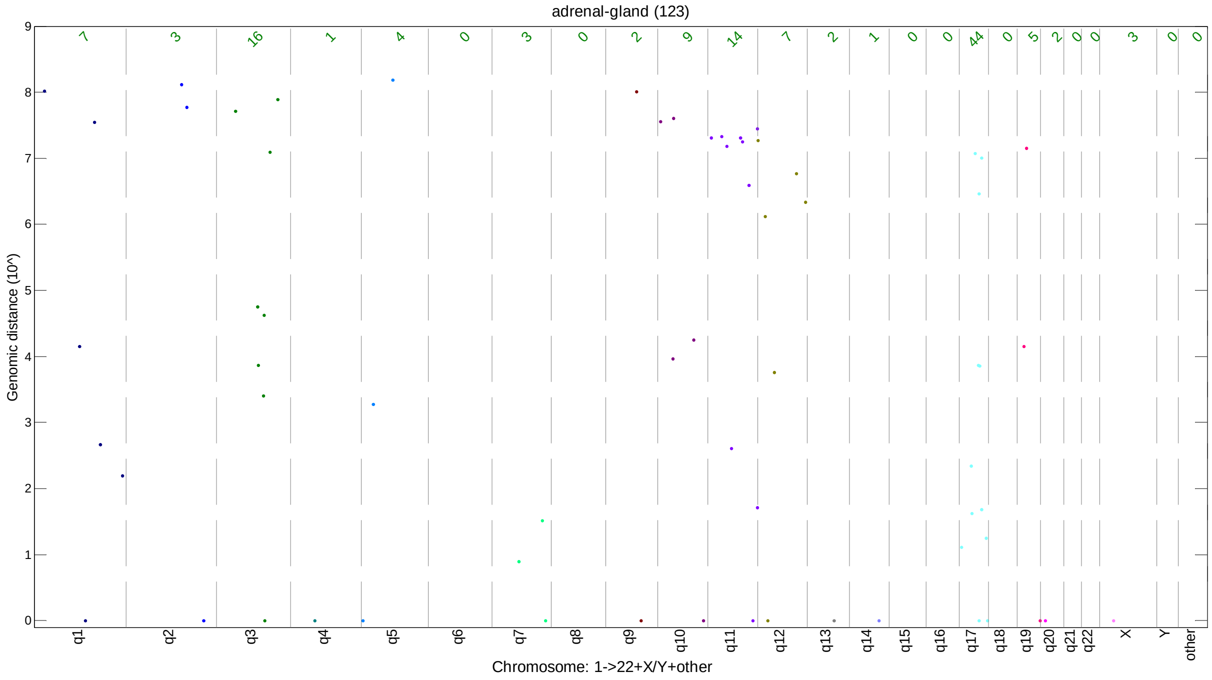

Figure S22 Mutation distribution across the chromosomes for 23 genome-wide screened adrenal gland tumor samples.

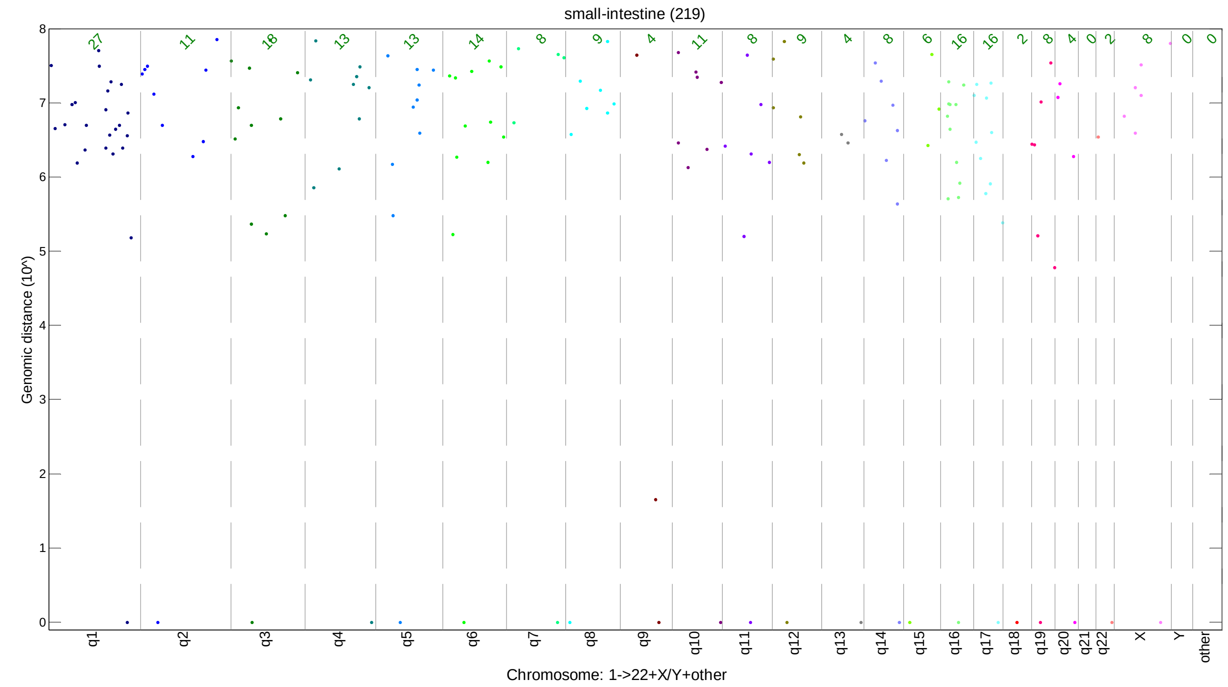

Figure S23 Mutation distribution across the chromosomes for 41 genome-wide screened small intestine tumor samples.

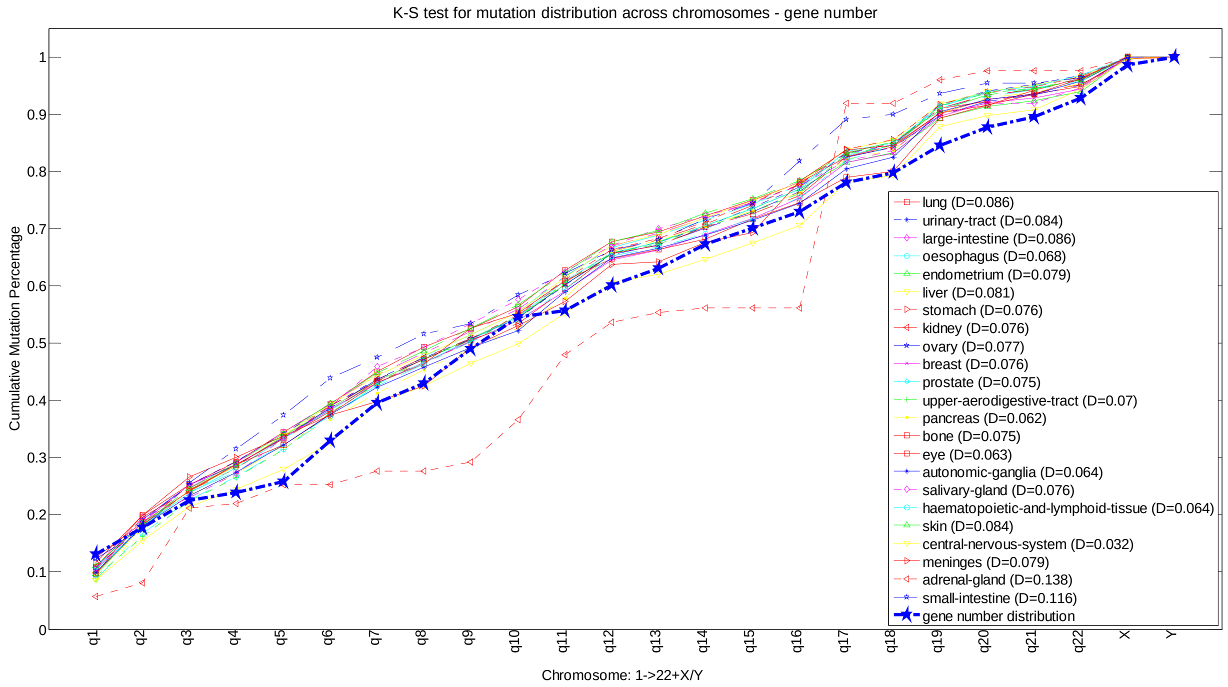

Figure S24 Kolmogorov-Smirnov test for the mutant gene number distribution across the chromosomes.

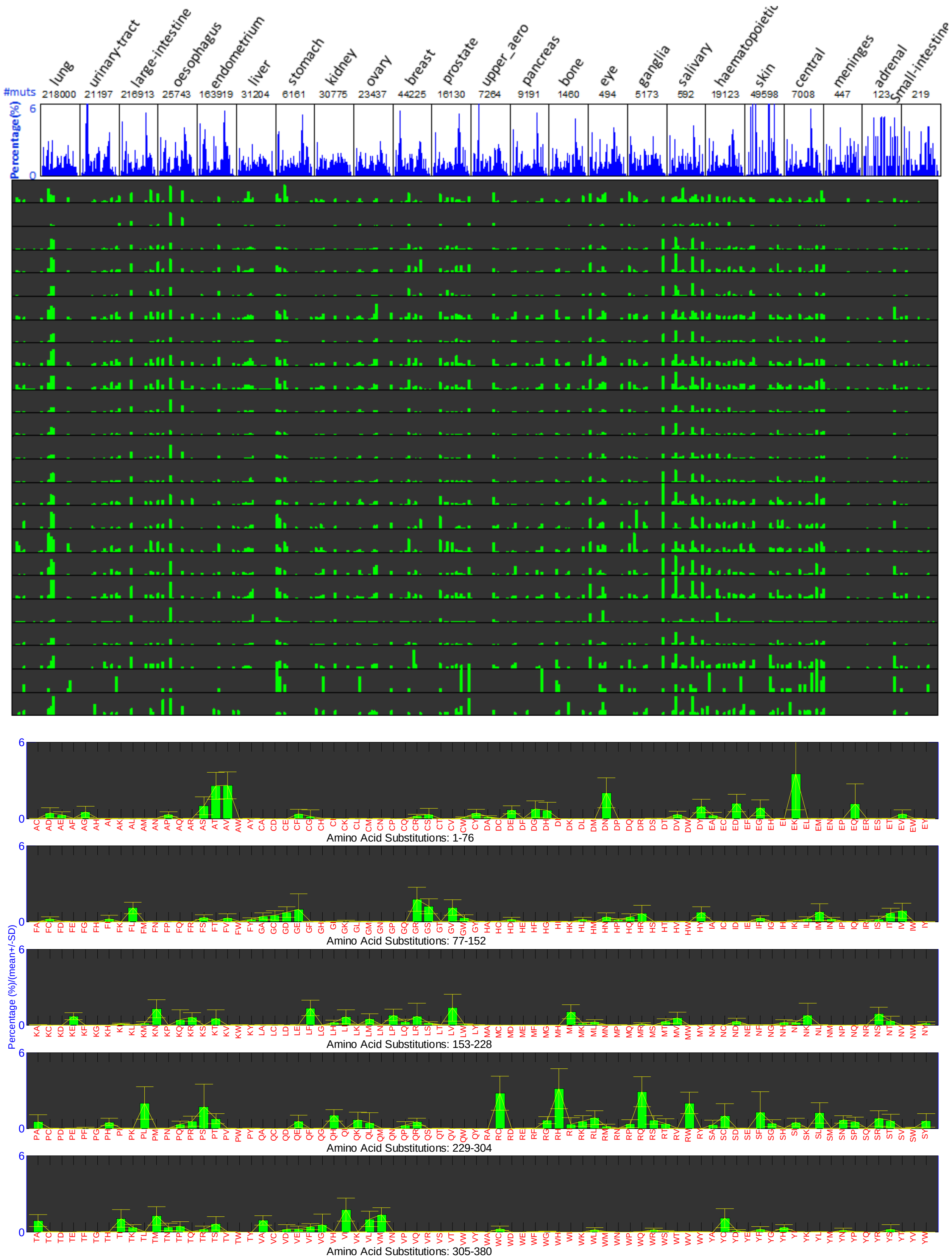

#muts
lung 218000
urinary-tract 21197
large-intestine 216913
oesophagus 25743
endometrium 163919
liver 31204
stomach 6161
kidney 30775
ovary 23437
breast 44225
prostate 16130
upper_aero 7264
pancreas 9191
bone 1460
eye 494
ganglia 5173
salivary 592
haematopoietic 19123
skin 49598
central 7008
meninges 447
adrenal 123
small-intestine 219
Percentage(%)
Percentage (%)/(mean+/-SD)
Amino Acid Substitutions: 1-76
Amino Acid Substitutions: 77-152
Amino Acid Substitutions: 153-228
Amino Acid Substitutions: 229-304
Amino Acid Substitutions: 305-380

Figure S25 Frequency distribution of missense mutations across the human chromosomes and frequency of 380 possible amino acid substitutions identified in 23 major human cancers. Top panel: the frequency distribution along the chromosomes, with the number of somatic mutations collected from COSMIC as denoted above each subfigure; middle panel: the frequency distribution along the 380 amino acid substitutions, each row corresponds to a cancer type ordered as the top panel and each green bar represents the percentage of that particular amino acid substitution in that cancer type. Bottom panel: a high-resolution representation of the horizontal axis of the middle panel, where the 380 possible residue substitutions are equally divided into four parts for clearer visualization. Each green bar stands for the average frequency of 23 cancers for that particular residue substitution. Standard deviations are also illustrated.

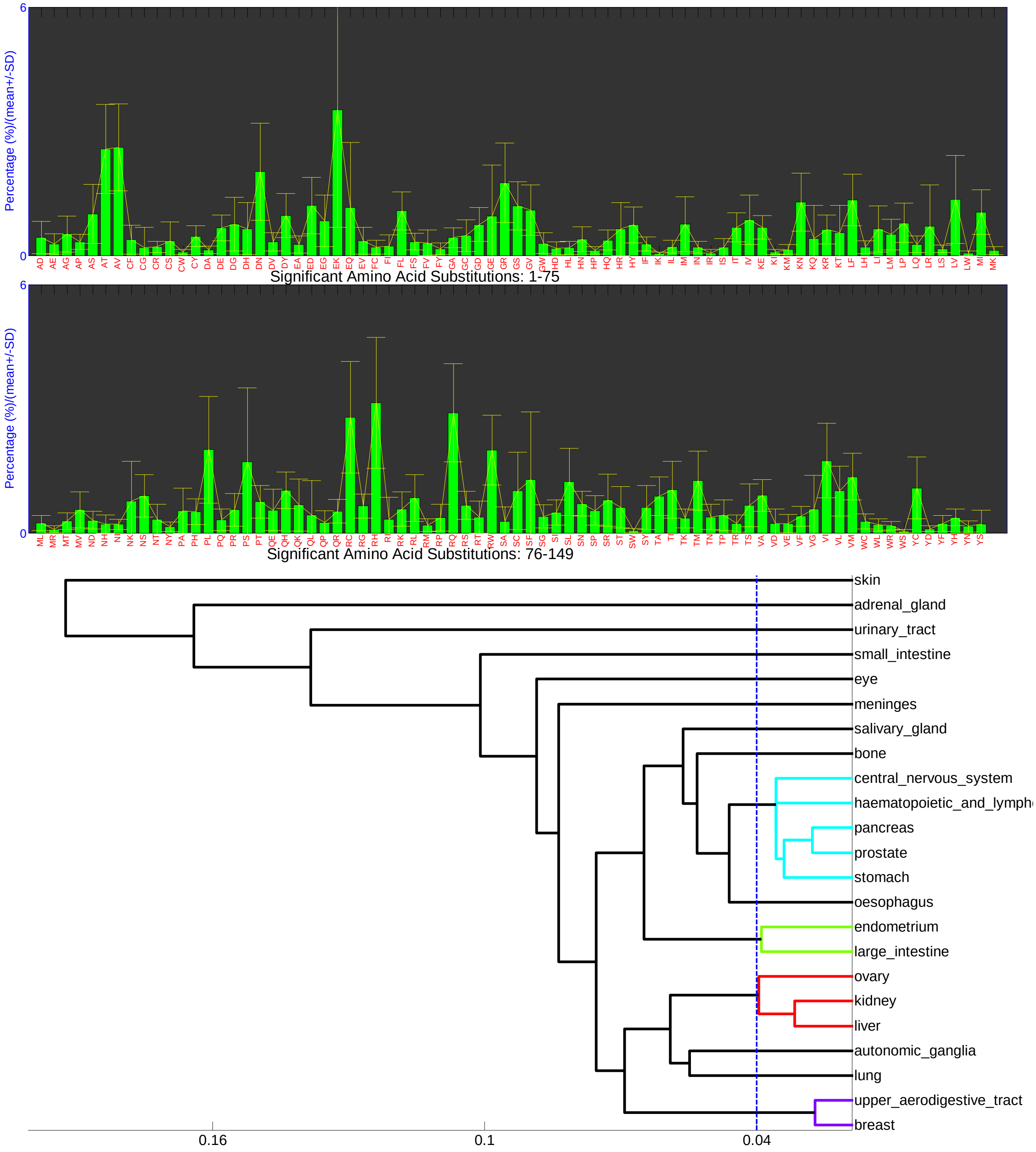

Figure S26 A higher resolution representation of average frequencies of 23 cancers at 149 significant amino acid substitutions with standard deviations (upper panel), and the dendrogram of the clustering results (lower panel) as shown in Figure 4.

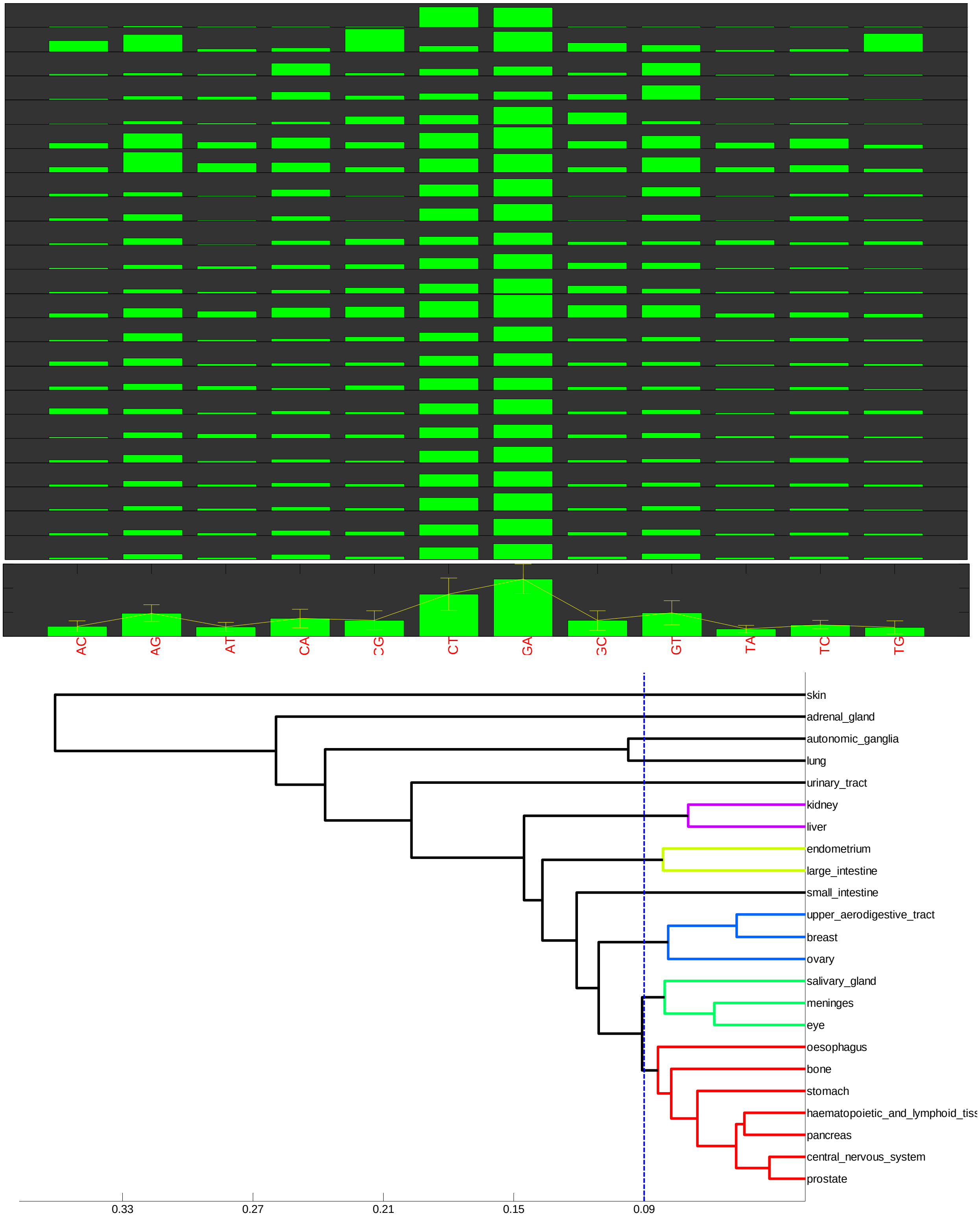

Figure S27 Mutation distribution along 12 nucleotide base pair changes for 23 major human cancers and the average of them (upper panel) and the clustering results according to their similarity in the mutation distribution (lower panel). The *y*-range for individual cancers is 0-50%, and for the average is 0-30%.

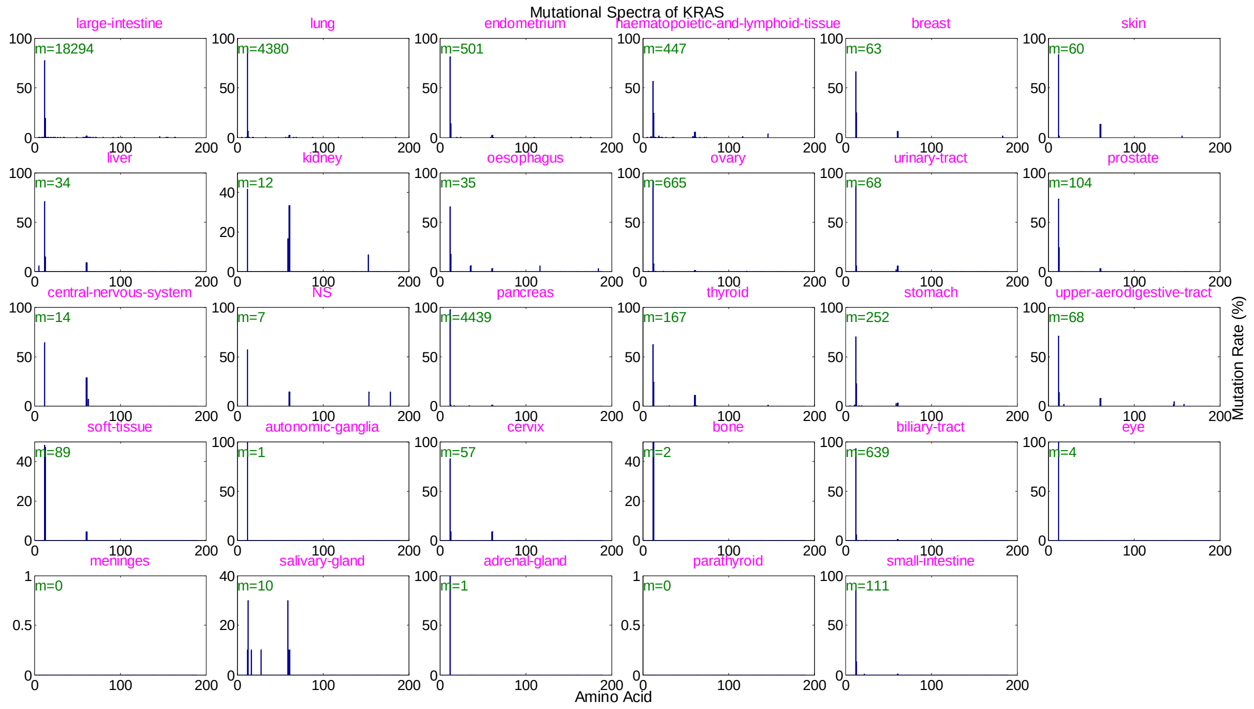

Figure S28 Mutational spectrum of the KRAS gene at the amino acid residue resolution.

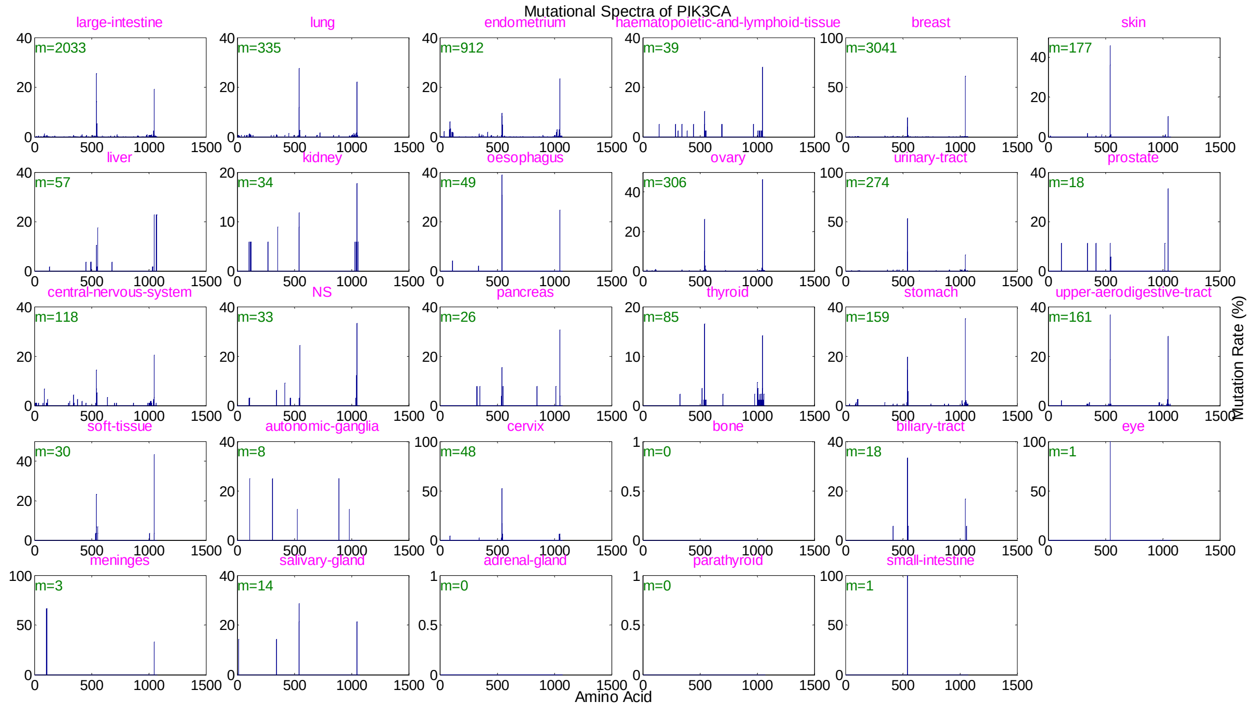

Figure S29 Mutational spectrum of the PIK3CA gene at the amino acid residue resolution.

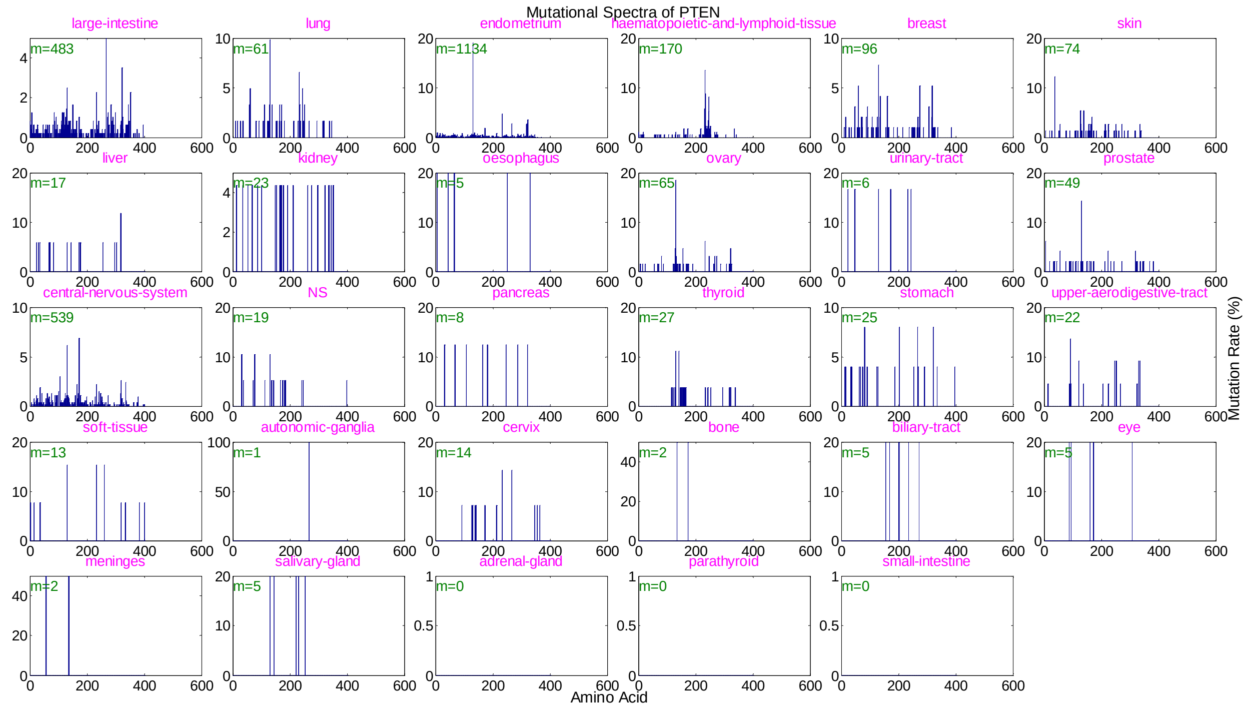

Figure S30 Mutational spectrum of the PTEN gene at the amino acid residue resolution.

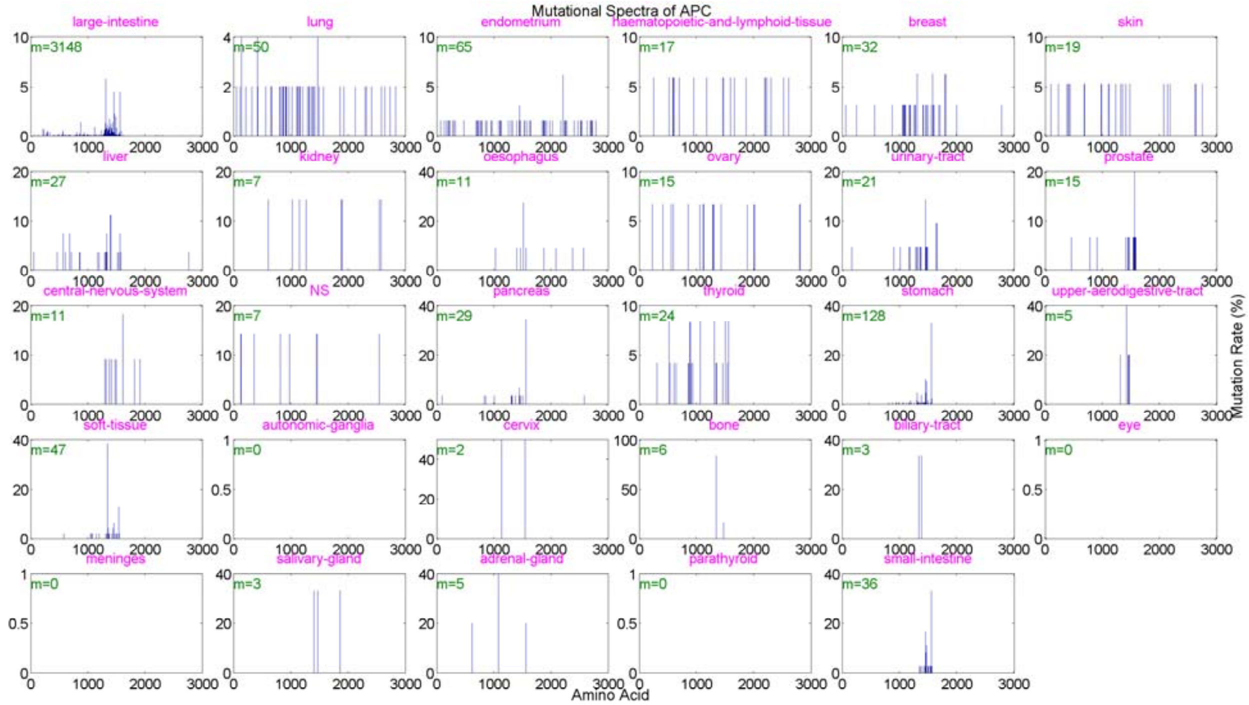

Figure S31 Mutational spectrum of the APC gene at the amino acid residue resolution.

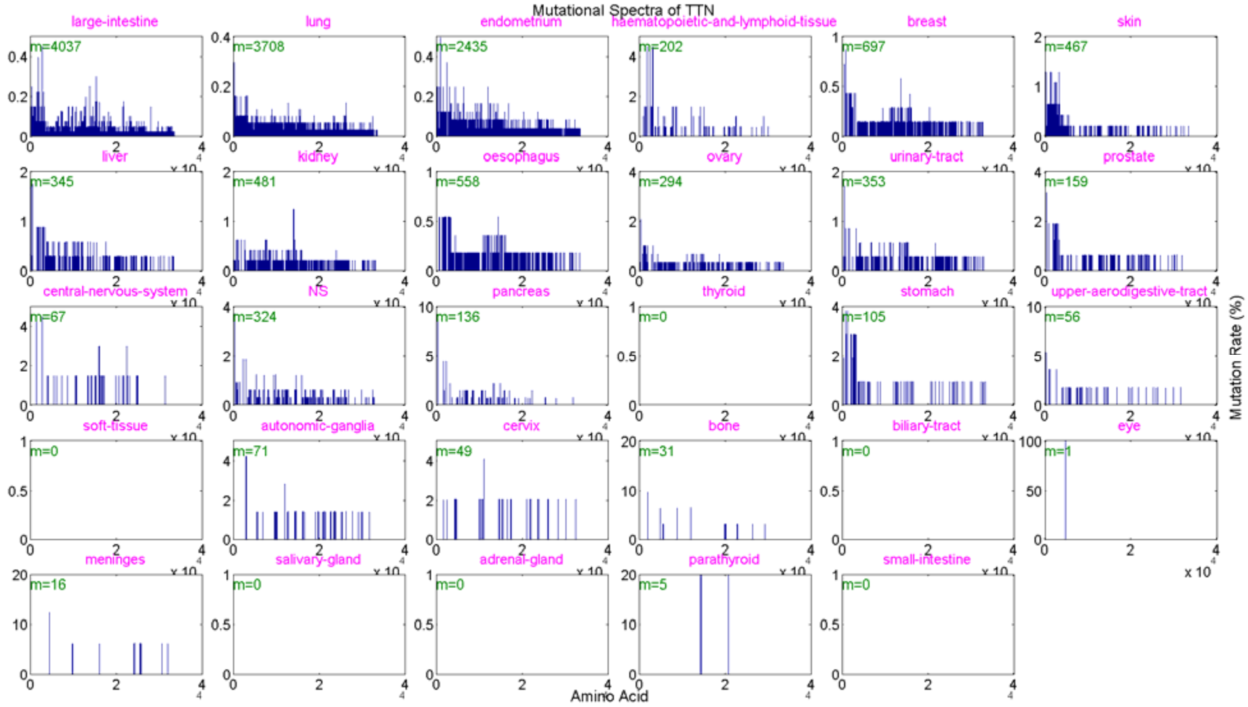

Figure S32 Mutational spectrum of the TTN gene at the amino acid residue resolution.

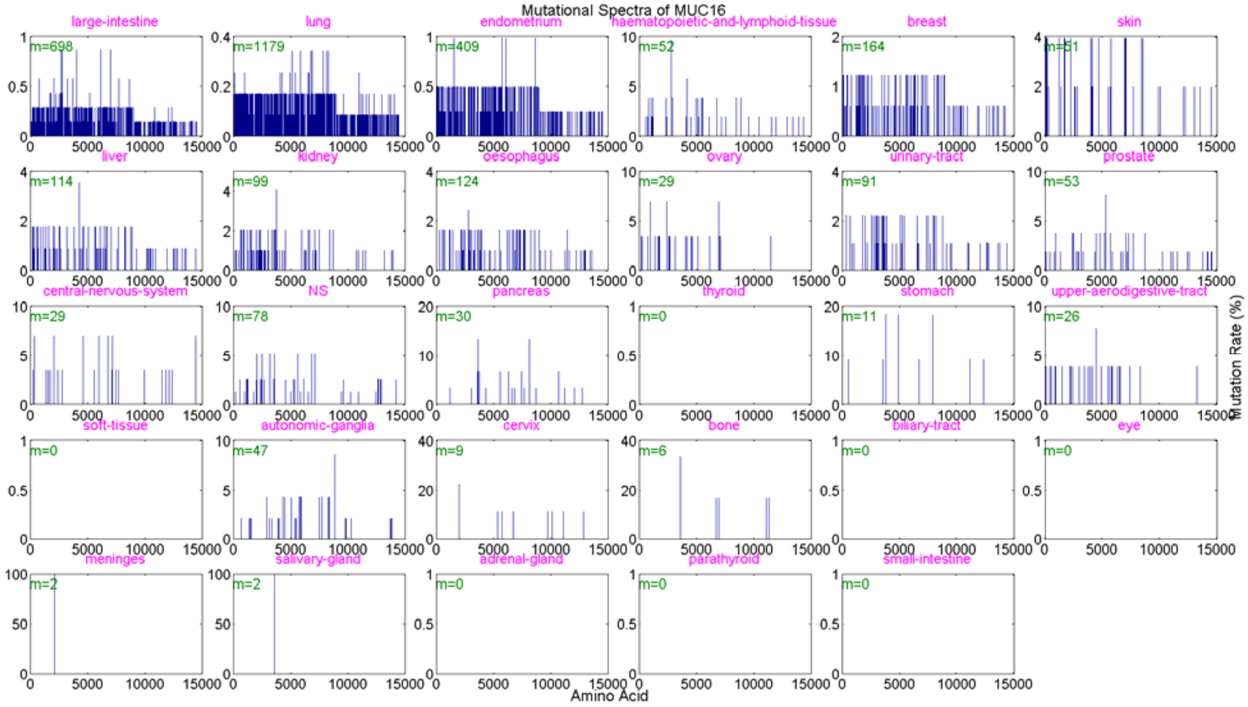

Figure S33 Mutational spectrum of the MUC16 gene at the amino acid residue resolution.

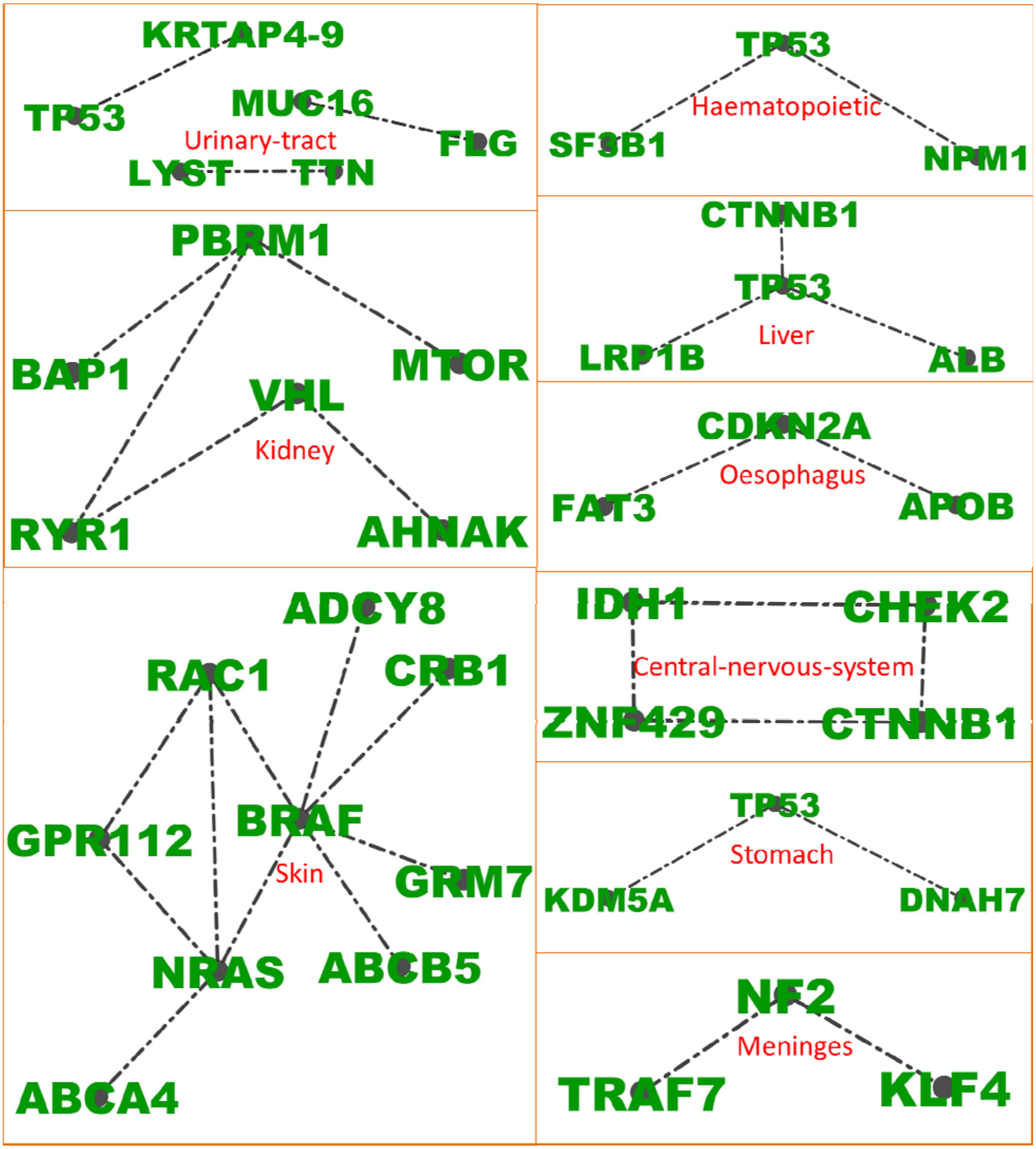

Figure S34 Gene pairs with significant exclusive pattern detected in 9 cancers as denoted in each sub-graph.

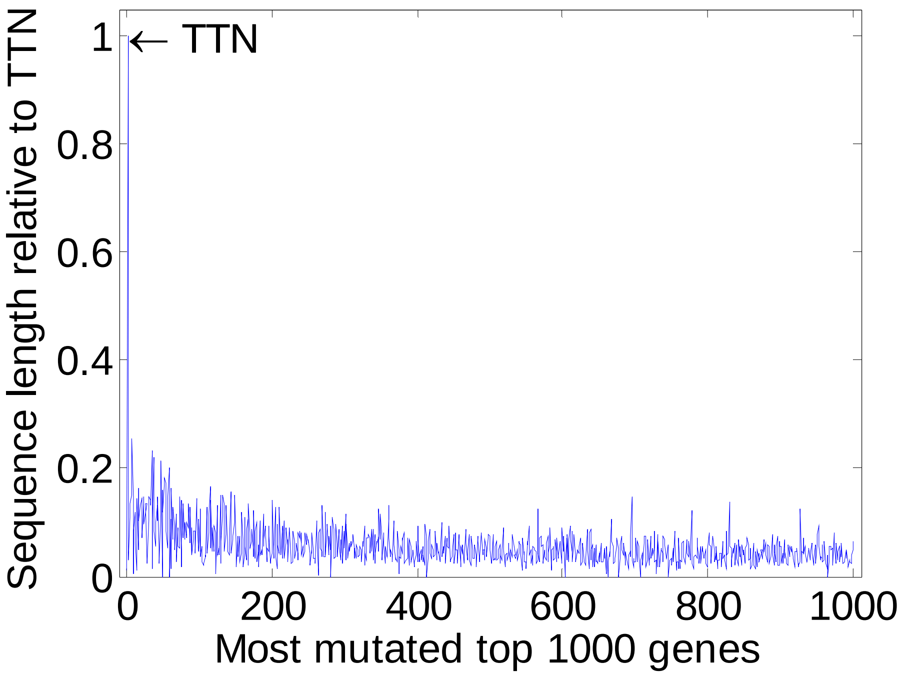

Figure S35 Protein sequence length relative to TTN (with 34350 amino acids) for the top 1000 frequently mutated genes detected in COSMIC v68. Protein sequence length was curated from UniProt (http://www.uniprot.org/). Only 990 out of the 1000 genes can be mapped to the current UniProt database, and the length of the remained 10 genes was left as 0.

Statistical significance analysis on cancer-specific mutation frequency of genes

We performed a statistical significance analysis on the top 1000 frequently genes (Table S2A) to explore their relative importance to specific cancer types. The significance for each gene is assessed based on the sample coverage and expected mutation frequency considering its sequence length. Suppose TTN (the longest protein) is mutated in a tumor sample with a probability of μ_{TTN}, under the null hypothesis that genes are mutated randomly determined only by their protein sequence length, the probability that an arbitrary gene *i* will be mutated in a tumor sample can be calculated by

$$\mu_i = \mu_{TTN} \cdot \frac{L_i}{L_{TTN}},$$

where L_i and L_{TTN} refer to the protein sequence length of gene *i* and gene TTN respectively. Since our purpose is to rank the relative importance of genes to a specific cancer, μ_{TTN} can be set as any reasonable positive value between 0 and 1. In our practice, we set μ_{TTN}=0.1, the order magnitude of which is comparable to the mutation frequency (/Mb) presented in (Lawrence et al., 2013, ref. 19 in main text). We obtained the sequence length by retrieving the genes in the UniProt protein database (http://www.uniprot.org/) and calculated the sequence length relative to TTN as shown in Figure S35. We applied our previously developed gene name – accession number correspondence table (Tan et al., 2012, ref. 22) to enable a maximum coverage of the 1000 test genes by UniProt. Since genes may have many aliases, most of them (495 out of 1000) could not be directly mapped to UniProt. Using the correspondence table, only 10 could not be mapped (denoted as 'NA' in Table S2C).

After obtained the background mutation probability of each gene, the statistical significance of each gene can be determined from the sample coverage profiles (Table S2A) by binomial test. For a specific cancer type *j*, if *K* out of N_j samples bears mutation(s) of gene *i*, the probability of observing $m \geq K$ samples with mutation(s) of gene *i* can be calculated as:

$$P_{ij} = P(\mathrm{m} \geq K) = \sum_{m=K}^{N_j} Bin(\mathrm{m} \mid \mathrm{N}_j, \mu_i) = \sum_{m=K}^{N_j} \binom{N_j}{m} \mu_i^{\,m} (1-\mu_i)^{N_j - m}$$

We first adopted this formula to generate Table S2C. Then, for each individual cancer, we sorted the 1000 *p*-values in increasing order. The 1000 *p*-values corresponded to the top 1000 frequently mutated genes listed in Table S2A. Genes with equal *p*-values are secondarily sorted according to their frequency detected in the current COSMIC (release 68), with more frequent genes ranking higher than less frequent ones. This ranking procedure produced supplementary Table S2D.

About the binomial *P*-value relating to arginine (*R*) substitution

In the results section we presented our statistical analysis result that arginine (*R*) turned out to be the most favorable target of amino acid alteration, in the sense that 17 out of the 23 major cancers carries at least one arginine substitution in their top 3 amino acid substitutions (Table 1, $P<10^{-9}$, binomial test). Here we elucidate how we figured out this significance level.

Under the null hypothesis that each of the 20 amino acids is randomly selected for a random mutation (substitution), for any specific cancer type, the probability that at least one arginine substitution ranks top 3 of all possible P(20,2)=20·19=380 amino acid substitutions can be calculated from the hyper-geometric distribution:

$$\mu = P(k \ge 1) = \sum_{k=1}^{n} \frac{\binom{K}{k}\binom{N-K}{n-k}}{\binom{N}{n}} = \sum_{k=1}^{3} \frac{\binom{19}{k}\binom{380-19}{3-k}}{\binom{380}{3}} = 0.143$$

Where K=19 refers to the number of possible arginine substitutions. Then the probability of observing this event happening in $m \geq 17$ out of N=23 cancer types can be computed from the binomial distribution:

$$\begin{aligned} P = P(\mathrm{m} \ge 17) &= \sum_{m=17}^{N} Bin(\mathrm{m} \mid \mathrm{N}, \mu) = \sum_{m=17}^{N} \binom{N}{m} \mu^{m} (1-\mu)^{N-m} \\ &= \sum_{m=17}^{23} \binom{23}{m} 0.143^{m} (1-0.143)^{23-m} \\ &= 1.8503 \times 10^{-10} \end{aligned}$$

It should be noted that this significance level was stated under the implicit assumption that all 20 amino acid residues are generally evenly distributed in nature. Interestingly, among the 20 amino acids arginine (*R*) is the only one that is much less frequently observed in nature than expected (King and Jukes, 1969). This implies that the predisposition of arginine mutation in human cancers is unlikely a purely random event, but has certain biological meaning.

Supplementary reference

Jack Lester King and Thomas H. Jukes. Non-Darwinian evolution. Science **16**, 788-798 (1969).
